# A Combinatorial Library of Lipid Nanoparticles for Cell Type‐Specific mRNA Delivery

**DOI:** 10.1002/advs.202301929

**Published:** 2023-04-24

**Authors:** Gonna Somu Naidu, Seok‐Beom Yong, Srinivas Ramishetti, Riccardo Rampado, Preeti Sharma, Assaf Ezra, Meir Goldsmith, Inbal Hazan‐Halevy, Sushmita Chatterjee, Anjaiah Aitha, Dan Peer

**Affiliations:** ^1^ Laboratory of Precision Nanomedicine The Shmunis School of Biomedicine and Cancer Research George S. Wise Faculty of Life Sciences Tel Aviv University Tel‐Aviv 69978 Israel; ^2^ Department of Materials Sciences and Engineering Iby and Aladar Fleischman Faculty of Engineering Tel Aviv University Tel Aviv 69978 Israel; ^3^ Center for Nanoscience and Nanotechnology Tel Aviv University Tel Aviv 69978 Israel; ^4^ Cancer Biology Research Center Tel Aviv University Tel Aviv 69978 Israel; ^5^ Nucleic Acid Therapeutics Research Center Korea Research Institute of Bioscience and Biotechnology (KRIBB) Chungcheongbuk‐do 28116 Republic of Korea

**Keywords:** cell type‐specific mRNA delivery, combinatorial lipid nanoparticles, mRNA delivery

## Abstract

Ionizable lipid‐based nanoparticles (LNPs) are the most advanced non‐viral drug delivery systems for RNA therapeutics and vaccines. However, cell type‐specific, extrahepatic mRNA delivery is still a major hurdle, hampering the development of novel therapeutic modalities. Herein, a novel ionizable lipid library is synthesized by modifying hydrophobic tail chains and linkers. Combined with other helper lipids and utilizing a microfluidic mixing approach, stable LNPs are formed. Using Luciferase‐mRNA, mCherry mRNA, and Cre mRNA together with a TdTomato animal model, superior lipids forming LNPs for potent cell‐type specific mRNA delivery are identified. In vitro assays concluded that combining branched ester tail chains with hydroxylamine linker negatively affects mRNA delivery efficiency. In vivo studies identify Lipid 23 as a liver‐trophic, superior mRNA delivery lipid and Lipid 16 as a potent cell type‐specific ionizable lipid for the CD11b^hi^ macrophage population without an additional targeting moiety. Finally, in vivo mRNA delivery efficiency and toxicity of these LNPs are compared with SM‐102‐based LNP (Moderna's LNP formulation) and are shown to be cell‐specific compared to SM‐102‐based LNPs. Overall, this study suggests that a structural combination of tail and linker can drive a novel functionality of LNPs in vivo.

## Graphical abstract

Herein, new ionizable lipid nanoparticles are developed and screened to enhance mRNA delivery in vivo. In vitro and in vivo screening identified two lipid‐based nanoparticles with novel functionality for in vivo mRNA delivery (Lipid 16 and Lipid 23). The combination of structural modules determines the novel functionality of ionizable lipids.

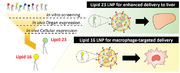

## 1 Introduction

Lipid nanoparticles (LNPs) have been the most advanced non‐viral drug delivery vectors for various RNA therapeutics since Patisiran (Onpattro) and mRNA vaccine approvals.^[^ ^1^ ^]^ The mRNA‐therapeutics deliver a protein‐encoding sequence of interest and have great potential for treating various diseases. Ionizable lipid‐based LNPs are the most advanced delivery vehicle for mRNA.^[^ ^1^, ^2^ ^]^ However, the majority of mRNA‐LNPs accumulate in the liver, and thus extra‐hepatic delivery is still considered a significant hurdle, especially for diseased cells within organs. Recent studies have demonstrated organ tropism of RNA‐LNPs to the spleen and lung by modifying the surface charge of LNPs.^[^ ^3^ ^]^ However, the current composition of the ionizable lipid nanoparticle formulation is well‐optimized for RNA loading and stability and modification of this formulation by adding, for example, a “selective organ targeting (SORT)” molecule or increasing the ratio of helper lipids could induce stability issues.^[^ ^3a,b^ ^]^

Cell type‐specific delivery of mRNA is the holy grail of therapeutics. A recent study reported that the replacement of helper lipid in LNP with an anionic lipid increases sinusoidal endothelial cell (SEC) delivery of mRNA in the liver,^[^ ^4^ ^]^ and targeting ligand‐modifications on the surface of LNPs enabled cell type‐specific mRNA delivery.^[^ ^5^ ^]^ However, adding a targeting moiety to the LNPs surface, such as a receptor‐binding antibody,^[^ ^6^ ^]^ via chemical conjugation of the ligand could be an unexpected challenge due to potential adverse effects on the stability of mRNA‐loaded LNPs, issues with controlling the orientation of the targeting ligand on the LNPs’ surface, as well as CMC for large scale production and clinical translation.

Rather than the charge‐ or ligand‐modification, the structure of ionizable lipid could drive an organ‐ and cell type‐tropism, for example, the piperazine structure‐bearing lipid for the broad immune cells population‐targeted mRNA delivery,^[^ ^7^ ^]^ and an organ‐selective mRNA delivery by the ester or amide bond‐containing tail structure (“O” or “N”‐series).^[^ ^8^ ^]^ These studies imply a probability of ionizable lipid structure‐driven, more precise cell type‐specific mRNA delivery. However, it hardly predicts the lipid function based on the lipid structure date.^[^ ^9^ ^]^

Our previous study described a library of lipid structures based on hydrazine, hydroxylamine, and ethanolamine linkers for siRNA delivery to leukocytes.^[^ ^10^ ^]^ Several of these lipids showed an efficient mRNA delivery in cancer cells, which outperformed MC3‐based LNP.^[^ ^2a^ ^]^ Yet, low mRNA delivery efficiency in vivo still hampers the development of novel mRNA therapeutics. Herein, eight new ionizable lipids were designed and synthesized using different hydrophobic tails and were screened to identify LNPs for enhanced mRNA delivery in vivo.

## 2 Results and Discussion

### 2.1 Structure of the Ionizable Lipids

**Figure 1:**
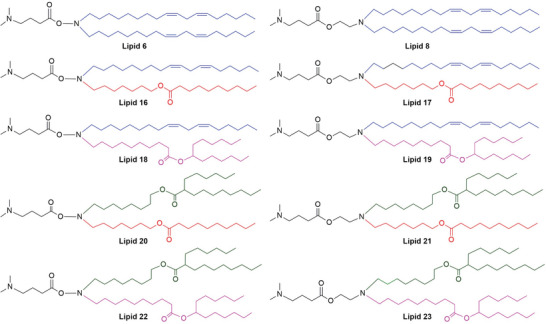
Structures of ionizable amino lipids. The lipids were synthesized with hydroxylamine/ethanolamine linkers with different hydrophobic tail chains. For Lipids 16 and 17, a linoleyl chain and a nonbranched ester chain were included as hydrophobic tail chains. Lipid 18 and Lipid 19 had linoleyl and branched ester tail chains. The non‐branched and branched ester tail chains were used for Lipid 20 and Lipid 21, whereas two different branched ester chains were used as tail chains for Lipid 22 and 23.

Our primary aim was to design and synthesize new amino ionizable lipids for enhanced mRNA delivery in vivo in a safe manner. In our previous study, we observed that hydroxylamine (Lipid 6) and ethanolamine (Lipid 8) linkers show good RNA delivery efficacy as compared to hydrazine linkers.^[^ ^10^ ^]^ Hence, Lipid 6 and Lipid 8 were used as reference pairs of lipids, wherein the linoleyl fatty acid chain used as both hydrophobic tails. Recently, the biodegradability of the tail was proved to be a critical factor for mRNA delivery in vivo,^[^ ^11^ ^]^ and a minor structural modification in the tails of ionizable lipids generated a novel functionality.^[^ ^8^ ^]^ Based on these studies, we rationally designed and synthesized a series of 8 new amino ionizable lipids by replacing the linoleyl chains of Lipids 6 and 8 with the other hydrophobic tails. For Lipids 16 and 17, the linoleyl chain was used as one hydrophobic tail, and the other hydrophobic chain was replaced with a non‐branched ester chain. In the case of Lipids 18 and 19, linoleyl and branched ester tails were used. The combination of non‐branched and branched ester tails was used for Lipids 20 and 21, whereas two different branched ester chains were used for Lipids 22 and 23 (**Figure** **1**). All the lipids were synthesized utilizing standard organic synthesis procedures and characterized by NMR and mass spectroscopic techniques (see Supporting Information).

### 2.2 In Vitro Screening of LNPs for Enhanced mRNA Delivery

**Figure 2:**
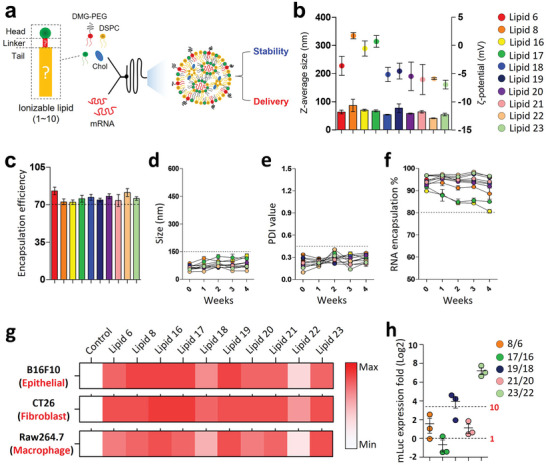
In vitro screening of amino ionizable LNPs for mRNA delivery. a) Schematic illustration of a microfluidic mixing preparation of mRNA‐encapsulated ionizable‐based LNPs b) Physicochemical characterization of mRNA‐encapsulated ionizable‐based LNPs. The size and *ζ*‐potential of mRNA‐encapsulated LNPs were measured using DLS. c) Encapsulation efficiency of mRNA in LNPs. Data are presented as mean ± S.D., *n* = 3–6/group for “b” and “c”. d‐f) Stability of mRNA‐encapsulated LNPs. DLS analysis of size distribution and PDI value at indicated days post LNP preparation. “d” for nano‐size, “e” for polydispersity index, “f” for RNA‐encapsulation (Percentile of LNP‐encapsulated mRNA to total RNA amount in the sample). Data are presented as mean ± S.E.M. g) Heat map image for an in vitro luciferase assay. 3‐different non‐hepatic cell lines were treated with mLuc‐LNP (mRNA dose: 0.2 µg mL^−1^), and the luciferase activity was measured 24hr post‐treatment. Data are presented as mean ± S.E.M., *n* = 3—6 per group for “d” to “g”. h) Ratio of in vitro luciferase activity between ionizable LNP pairs. The luciferase activity ratio between LNP pairs with the ethanolamine‐linker and the hydroxylamine‐linker is shown. Each dot indicates the mean value from each cell line. Data is presented as mean ± S.E.M.

Ionizable lipid based‐LNPs were prepared using a microfluidic mixing device, Nanoassemblr, as previously described^[^ ^2^, ^12^ ^]^ (**Figure** **2**a). All LNPs encapsulating the firefly luciferase mRNA (mLuc) showed homogeneous nano‐size distribution measured between 40 and 80 nm in diameter and *ζ*‐potential, measured between −7 to 2 mV. Each pair of LNPs show similarity in its physicochemical characteristics (Figure 2b). Encapsulation efficiency was evaluated as >70% for all the LNP formulations (Figure 2c). DLS analysis of mRNA‐encapsulated LNPs revealed that the size, PDI, and RNA encapsulation were stable for more than a month in a regular refrigerator (2–8 °C). One pair of LNPs prepared with Lipid 16 and 17 was slightly less durable than the others. These two lipids include a non‐branched ester chain in one of the hydrophobic tails (Figure 2d–f). Although in vitro delivery efficiency of mRNA‐LNPs does not always correlate with in vivo delivery efficiency,^[^ ^13^ ^]^ in vitro assay could be used as a selection strategy to exclude LNPs with a low mRNA delivery efficiency of optimized formulations.^[^ ^13^, ^14^ ^]^ To this end, the mRNA delivery efficiency of these different LNPs formulations was evaluated in 3 different, non‐hepatic cell lines, B16F10, CT26, and Raw264.7, representing epithelial, fibroblast, and macrophage cells, respectively. In vitro luciferase assay identified 2‐ionizable lipids with lower mRNA delivery efficiency in all three cell lines, with the most inadequate mRNA delivery by Lipid 22, which shows more than two orders of magnitude lower firefly luciferase expression in comparison with that of Lipid 16 (Figure 2g, Figure S1b, Supporting Information). The ionizable lipids with hydroxylamine linker showed less mRNA delivery efficiency than their ethanolamine linker pairs, especially for Lipids 18 and 22, which have branched ester chains in their structure. The pair of lipids with branched ester tails showed the most significant difference in between for luciferase mRNA delivery (Figure 2h). Finally, it was concluded that combining hydroxylamine linker with branched ester fatty acid tail chains led to lower mRNA delivery, and 2 ionizable lipids, 18 and 22, were excluded from the following in vivo screening experiments.

### 2.3 In Vivo Screening of LNPs for mRNA Delivery

**Figure 3:**
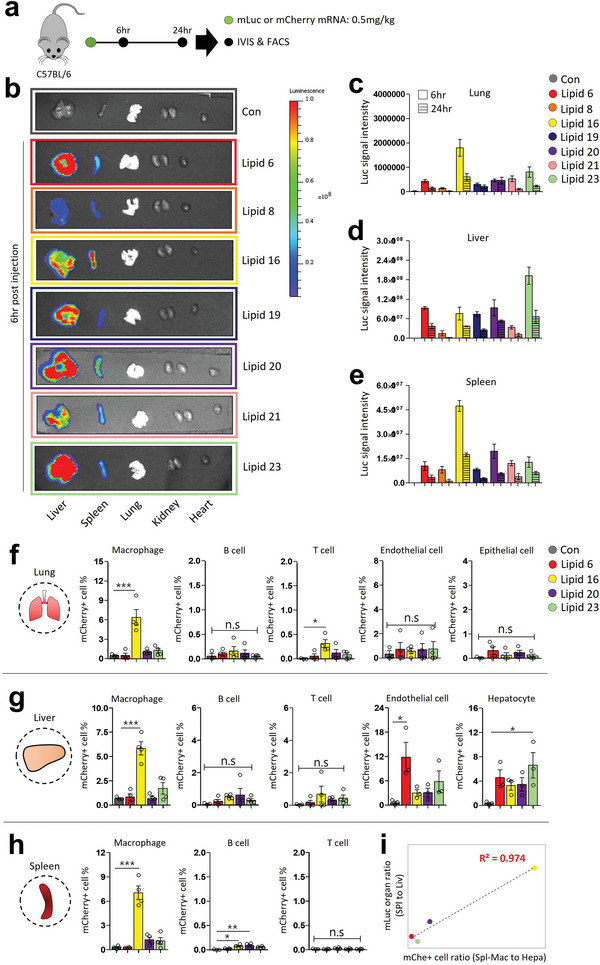
In vivo screening of amino ionizable LNPs for mRNA delivery. a) Experimental scheme for in vivo screening study. The mLuc or mCherry mRNA‐encapsulated LNPs (mRNA dose: 0.5 mg kg^−1^) were injected into wild‐type C57BL/6 mice through the tail vein, and organ and cellular expression were analyzed at 6, 24hr postinjection. b) Representative organ images for in vivo luciferase assay. Mice were injected with D‐luciferin (150 mg kg^−1^), and major organs were harvested at 6 h post mRNA injection. c‐e) Bar graph for in vivo luciferase assay. The in vivo luciferase activity for organs was shown at 6 and 24 h postinjection. Bar and lined bar indicate 6 and 24 h results, respectively. The “c”, “d”, and “e” represents the lung, liver, and spleen results. f‐h) Bar graph for in vivo cellular distribution study. Mice were injected with mCherry mRNA‐encapsulated LNPs, and mCherry‐expression was analyzed using flow cytometry at 6 h post‐injection. i) The Correlation study for organ luciferase activity and cellular mCherry‐expression. The correlation between a luciferase activity ratio (spleen to the liver) and a mCherry‐expression ratio (CD11b^−hi^ splenic macrophage to hepatocyte) was shown for each LNP. Data are presented as mean ± S.E.M, *P < 0.05, **P < 0.01, ***P < 0.001 by one‐way ANOVA with Tukey's post hoc test, n.s = not significant, *n* = 3–5 mice per group for “c” to “e”. *n* = 3–4 mice per group for “f” to “h”.

To screen the remaining 8 ionizable lipids in vivo, wild‐type C57BL/6 mice were injected with mRNA‐encapsulated LNPs through the tail vein, and the mRNA expression was analyzed at the organ‐ and cellular‐ levels (**Figure** **3**a). mLuc was delivered by the LNPs, and in vivo luciferase activity was measured to compare in vivo mRNA delivery between the different LNP formulations. The 0.3–1.0 mg kg^−1^ is the generally accepted dose for the systemic administration of mRNA in vivo.^[^ ^5^, ^7^, ^8^, ^15^ ^]^ To this end, we injected the mice with an mRNA dose of 0.5 mg kg^−1^. At 6hr postinjection, most of the luciferase signal was detected in the liver and the spleen for all LNPs, which are the major organs of LNPs accumulation^[^ ^3a,b^ ^]^ (Figure 3b). Lipid 8, one of the reference lipids, showed the lowest luciferase expression in the organs. As presented in the organ luciferase signal bar graphs, two lipids show a noticeable difference compared to a reference lipid, Lipid 6. We observed higher luciferase expression in the lungs (4.18‐fold) and spleen (4.56‐fold) by Lipid 16‐LNP compared to Lipid 6‐LNP. Lipid 20‐LNP showed slightly higher mRNA delivery to the spleen than Lipid 6‐LNP (Figure 3c–e). Lipid 23‐LNP showed the highest mRNA delivery in the liver (2.03‐fold), which was higher than Lipid 6‐LNP (Figure 3c–e). Although Lipid 17‐LNP showed a comparable luciferase expression to Lipid 16‐LNP in vitro, the lowest organ luciferase expression was observed in Lipid 17‐LNP and therefore was excluded from the experimental set of 24 h (Figure S2, Supporting Information). The “Spleen to liver” ratio of luciferase signal intensity indicated the highest value for LNPs formed by Lipid 8 and Lipid 16, while the lowest values were observed with the Lipid 23‐LNP group (Figure S3, Supporting Information).

As previously reported,^[^ ^16^ ^]^ cell type‐tropism of mRNA‐LNPs drives the extra‐hepatic organ distribution, especially towards the spleen. To this end, mCherry mRNA‐encapsulating LNPs were systemically injected, and mRNA expression was analyzed at cellular levels to address the cell type‐specificity of LNPs. Lipids 6, 16, 20, and 23 were chosen based on the in vivo luciferase expression study for this cellular distribution study. The mCherry mRNA‐LNPs showed a size distribution ranging from 50 to 80 nm in diameter, which is in line with the preparations of mLuc‐LNPs (Figure S4, Supporting Information). The cells were gated based on the expressions of CD45, CD11b, Gr1, F4/80, CD3, CD19, and CD31. The CD45^+^CD11c^mid/low^CD11b^hi^F4/80^+^Gr1^mid/low^ cells were gated as CD11b^hi^ macrophages.^[^ ^17^ ^]^ CD45^+^CD3^+^ and CD45^+^CD19^+^ cells were gated as T and B cells, respectively. The CD45^−^CD31^+^ and CD45^−^CD31^−^ cells were identified as endothelial and hepatocyte/epithelial cells, respectively (Figure S5, Supporting Information).^[^ ^2^, ^18^ ^]^ Lipid 6‐LNPs exhibited most of the mCherry‐expression in endothelial cells (11.85%) and hepatocytes (4.57%) in the liver, but no significant expression was observed in other types of cells and organs. Lipid 23‐LNP showed a greater mCherry‐expression in hepatocytes (6.61%) and CD11b^hi^ macrophages (1.72%) than others. Interestingly, Lipid 16‐LNP showed the highest mCherry‐expression in the CD11b^hi^ macrophages in all organs (6.38%, 5.84%, and 7.04% for mCherry^+^CD11b^hi^ macrophages in the lung, liver, and spleen, respectively) while slightly lower mCherry‐expression in hepatocytes and liver endothelial cells in comparison with the Lipid 6 and 23 LNPs (Figure 3f–h, Figure S6, Supporting Information). A high correlation (R^2^: 0.974) was observed between the “mCherry‐expression ratio (splenic CD11b^hi^ macrophages to liver hepatocytes)” and “mLuc‐expression ratio (spleen to the liver)” (Figure 3i). However, the size or charge of LNPs did not show a significant correlation with splenic mRNA delivery, which is distinguished from the negatively charged LNPs for splenic mRNA delivery in the previous study (Figure S7, Supporting Information).^[^ ^3a,b^ ^]^ A lower pKa value of LNP was identified as a factor increasing the splenic mRNA delivery.^[^ ^19^ ^]^ A result of the TNS assay indicated a low correlation (*R* ^2^: 0.3) between the pKa value of mRNA‐LNPs and the splenic mRNA expression level (Figure S8, Supporting Information). These results support our claim that the mechanism for splenic mRNA delivery of Lipid 16‐LNP is different from the previously reported mechanisms of charge‐ and pKa‐ mediated splenic delivery.^[^ ^16^, ^19^ ^]^ Protein corona on the surface of LNPs was identified as a critical factor for an alteration of organ selectivity.^[^ ^8^, ^9^, ^19^ ^]^ Besides the characteristic of LNP, a difference in the intracellular environment in macrophages could be an inducer for the cell type tropism.^[^ ^20^ ^]^ Further details on the mechanism for the cell type‐specificity of Lipid 16‐LNP, such as the protein corona composition, should be addressed in future studies. In conclusion, Lipid 23 was identified as a superior lipid for liver mRNA delivery, and Lipid 16 was identified as a cell type‐specific lipid for CD11b^hi^ macrophages. The higher luciferase expression of Lipid 16‐LNP was observed in the spleen and lungs might be derived from the macrophage‐specific mRNA expression in these organs, as shown by our preferential expression of mCherry in CD11b^hi^ macrophages.

### 2.4 In Vivo Selective and Tumor‐targeted mRNA Delivery via LNPs

**Figure 4:**
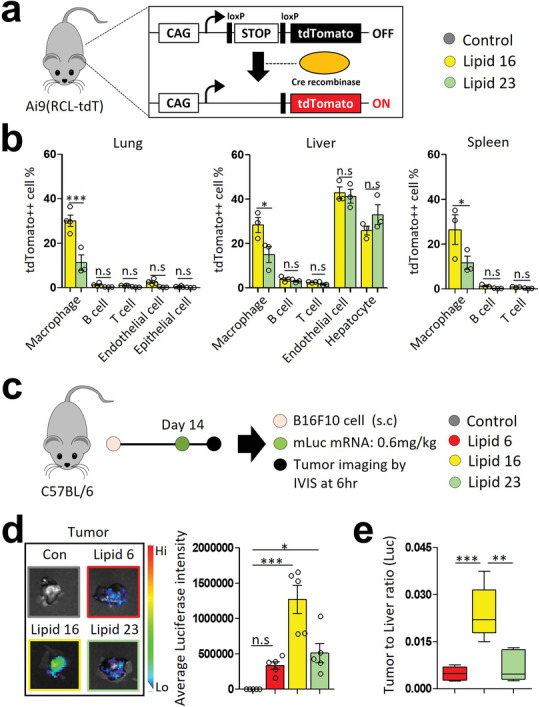
In vivo Cre mRNA delivery & tumor‐targeted mRNA delivery study. a) Experimental scheme for Cre mRNA delivery in vivo. The Ai9 (RCL‐tdT) mice were injected with Cre recombinase mRNA‐LNPs (mRNA dose: 0.6 mg kg^−1^), and the organs were harvested at 72 h post‐injection. b) Bar graph for the ratio of tdTomato‐expressing cell. The organs were digested into single cells for flow cytometric analysis to measure tdTomato‐expression in various cell types. c) Experimental scheme for tumor‐targeted mRNA delivery study. B16F10 tumor‐bearing mice were intravenously injected with mLuc‐LNPs (mRNA dose: 0.6 mg kg^−1^), and tumors were harvested at 6hr post‐injection to measure luciferase intensity. d) Representative tumor images and bar graph of luciferase signal intensity. e) Bar graph for tumor‐to‐liver luciferase intensity ratio. Data are presented as mean ± S.E.M for “b” to “d” and Box & Whiskers for “e”, *P < 0.05, **P < 0.01, ***P<0.001 by one‐way ANOVA with Tukey's post hoc test, n.s = not significant, *n* = 3–4 mice per group for “b”, *n* = 5 mice per group for “d, e”.

Although the mLuc and mCherry mRNA delivery studies represent a temporal expression of mRNA delivered by the LNPs, it is hard to analyze an accumulative effect of mRNA expression by these kinds of surrogate markers. Hence, a cumulative effect of mRNA expression was investigated in the tdTomato‐Cre recombinase mouse model.^[^ ^21^ ^]^ The Cre recombinase mRNA‐encapsulated, Lipid 16‐ and 23‐ LNPs showed homogeneous size distribution (Figure S9, Supporting Information). The Cre recombinase‐induced tdTomato‐expression was analyzed in the organs at 72 h post intravenous injection (**Figure** **4**a). As previously reported, extracellular vesicles transport cellular mRNA and proteins to the neighboring cells.^[^ ^21b^ ^]^ Hence, the tdTomato^−hi^ population was gated as the Cre mRNA‐delivered cells (Figure S10, Supporting Information). Both LNPs exhibit the highest tdTomato‐expression in the liver endothelial cells and hepatocytes with ≈40% expression level. A slight difference between Lipid 16 and 23 was shown for hepatocyte expression (1.27‐fold). A significant difference in tdTomato‐expression between Lipid 16 and 23 was observed in the CD11b^hi^ macrophage population in all organs. These results align with the mCherry mRNA delivery data (Figure 4b, Figure S11, Supporting Information).

As reported in our previous study,^[^ ^12^ ^]^ the CD11b^hi^ macrophages consist of tumor myeloid/immune cells. We hypothesized that the CD11b^hi^ macrophage‐tropism of Lipid 16 would increase the mRNA delivery to a solid tumor. We, therefore, employed the B16F10 melanoma model to address this hypothesis (Figure 4c). Flow cytometric analysis identified ≈4% of the CD11b^hi^ macrophages in a B16F10 tumor tissue, which is the comparable frequency in lung tissue (Figure S12, Supporting Information). Lipid 16‐LNP showed a higher luciferase mRNA delivery for tumor tissue compared to Lipid 6‐ and Lipid 23‐ LNPs with 3.79‐ and 2.48‐fold increased intensity of luciferase signal (Figure 4d). The tumor‐to‐liver ratio of luciferase intensity indicated the highest value for Lipid 16‐LNP (Figure 4e). These results suggest a potential of Lipid 16‐LNP for tumor‐targeted therapeutic mRNA delivery, such as a secretory immune checkpoint blockade antibody and anti‐tumoral cytokine. In addition, the mRNA delivery potency of Lipid 16‐ and 23‐ LNPs was evaluated for a month. Both LNPs showed a decrease in mRNA delivery efficiency depending on the time. A sharper decline of mRNA delivery efficiency was observed for Lipid 23‐LNP than that of Lipid 16‐LNP with 50% and 68% in CT26 cells at four weeks after LNP‐preparations, respectively (Figure S13, Supporting Information).

### 2.5 No In Vivo Toxicity Observed for mRNA‐LNPs

**Figure 5:**
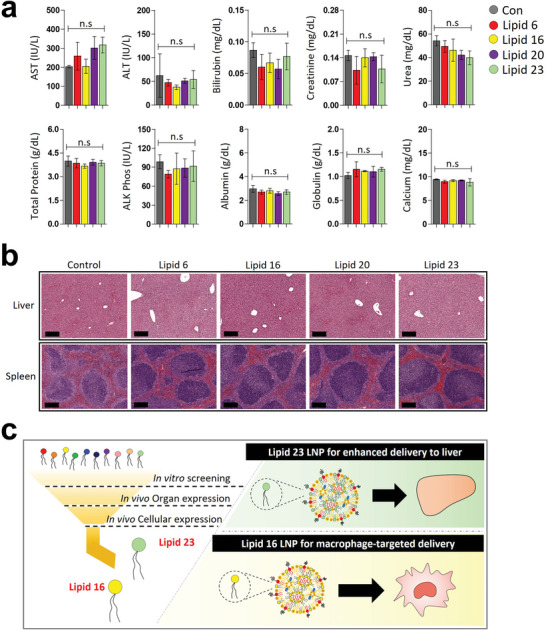
No in vivo toxicity of mRNA ‐LNPs. a) Whole blood was harvested at 24 h post‐mLuc‐LNP injection, and levels of liver enzymes, creatinine, bilirubin, and total protein, were measured. Data are presented as mean ± S.D, analyzed by one‐way ANOVA with Tukey's post hoc test, n.s = not significant, *n* = 3 mice per group. b) Representative tissue histology images. Spleen and liver were harvested for H&E staining at 24 h post‐mLuc‐LNP injection (mRNA dose: 0.5 mg kg^−1^). The black bar indicates 200 µm. c) Scheme for an experimental summary.

Finally, in vivo toxicity of mRNA‐LNPs was evaluated by measuring liver enzyme levels and organ histology. No significant differences in the liver enzyme levels and blood markers such as creatinine, bilirubin, and total protein levels were observed for all LNP‐treated groups compared with the control group at the indicated mRNA dose, 0.5 mg kg^−1^ (**Figure** **5**a). H&E staining images showed no signs of toxicity for the liver and spleen at 24 h post‐mRNA‐LNP injection (Figure 5b).

### 2.6 Comparison Study with SM‐102 LNP for mRNA Delivery In Vivo

**Figure 6:**
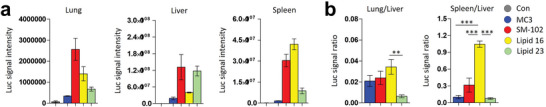
Comparison of the mRNA‐LNPs with SM‐102 LNP for mRNA delivery in vivo. a) Bar graph for mRNA delivery in organs. C57BL/6 mice were injected with mLuc‐LNPs, and luciferase intensity was measured at 6hr post injection (mRNA dose: 0.5 mg kg^−1^). b) Bar graph for organ luciferase intensity ratios. Data are presented as mean ± S.E.M., **P < 0.01, ***P < 0.001 by one‐way ANOVA with Tukey's post hoc test, mice *n* = 3—5 per group.

SM‐102 is the ionizable lipid used for developing an mRNA vaccine against COVID‐19 and is approved for mRNA delivery in humans.^[^ ^15^, ^22^ ^]^ We compared the mRNA delivery efficiency of our LNPs (Lipids 16 and 23) with SM‐102 LNP via organ expression, immunogenicity, and toxicology studies. SM‐102 LNP showed a comparable mRNA delivery with Lipid 23‐LNP for the liver. Lipid 16‐LNP showed a higher mRNA delivery to the spleen but lower mRNA delivery to the lung compared to SM‐102 LNP (**Figure** **6**a). Lipid 16‐ and Lipid 23‐LNPs showed the highest‐ and lowest‐ values for the organ luciferase intensity ratios (lung/liver and spleen/liver). Conclusively, the systemic mRNA delivery efficiency of our LNPs (Lipid 16, Lipid 23) is comparable with SM‐102 LNP but shows a higher specificity for each organ (Figure 6b). To date, mRNA therapeutics have been applied to mice models in various ranges of doses from 0.3 to 1.0 mg kg^−1^.^[^ ^5^, ^7^, ^8^ ^]^ Tolerability for repeated administration is one of the requirements for the therapeutic application of LNPs.^[^ ^23^ ^]^ Lipid 16, Lipid 23, and SM‐102 LNPs were systemically administered and evaluated for mRNA expression to address the tolerability of mRNA‐LNPs in 2 different doses, 0.25 and 1.0 mg kg^−1^. There was no significant difference between 1st and 2nd dosing for the mRNA expressions of all LNPs in the whole‐body bioluminescence level (Figure S14, Supporting Information). Lastly, we compared the toxicity and immunogenicity of the LNPs with SM‐102 LNP by measuring liver enzymes and blood cytokine levels at two different doses, 0.5 and 1.0 mg kg^−1^. At 3hr postinjection, all LNP‐injected groups showed increased cytokine levels of MCP1 and IL6 compared to the control group. And higher blood levels of MCP1 and IL6, not for the C3 complement factor, were shown for the Lipid 16‐LNP group compared with that of Lipid 23‐ and SM‐102‐ LNPs at the indicated dose of 0.5 mg kg^−1^. However, all LNPs showed comparable cytokine levels at the 1.0 mg kg^−1^ dosage. All cytokine levels declined to the normal range of the control group at 24 h postinjection regardless of the dose (Figure S15a, Supporting Information). The liver enzymes and blood chemistry did not significantly differ between all LNPs at the indicated doses, 0.5 and 1.0 mg kg^−1^ (Figure S15b, Supporting Information). Our results demonstrate a comparable immunogenicity/toxicity of our LNPs with SM‐102 LNPs.

## 3 Conclusions

Herein, a novel amino ionizable lipids library was synthesized and vigorously screened to identify the best lipids for efficient mRNA delivery in vivo. The physicochemical characterization study validated the homogeneous preparation of all ionizable LNPs. The in vitro luciferase assay was used to exclude the lipids with low mRNA delivery efficiency for non‐hepatic cells. The result showed that the lipids with branched ester tail chains have a lower mRNA delivery efficiency when combined with the hydroxylamine linker. The mLuc‐expression in vivo exhibited different organ distribution between the LNPs. The cellular distribution study using mCherry mRNA revealed that one ionizable lipid (Lipid 16) had CD11b^hi^ macrophage‐tropism for mRNA delivery. Moreover, splenic expression of Luciferase mRNA was correlated with macrophage‐specific expression of mCherry mRNA, which suggests that the spleen‐ and lung‐ expression of mRNA might be derived from the macrophage‐tropism of Lipid 16 in these organs. The macrophage‐specificity of Lipid 16 was validated again in the Cre‐Lox model. In vivo study in B16F10 tumor‐bearing mice showed a higher mRNA delivery effect of Lipid 16 for tumors, which was explained by the CD11b^hi^ macrophages in the tumor microenvironment. On the mechanism of cell type‐specificity, there was no significant correlation between the splenic mRNA delivery effect of Lipid 16 and the previously reported factors such as pKa value, size, and surface charge of LNPs.^[^ ^3^, ^19^ ^]^ Further investigations, such as the corona protein composition of LNPs and intracellular trafficking, should be addressed to understand the mechanism of this CD11b^hi^ macrophage‐tropism. The in vivo studies with SM‐102 LNP indicated a comparable mRNA delivery efficiency of our LNPs upon systemic injection with higher organ specificities. Although our results demonstrate that the CD11b^hi^ macrophage population is the primary cell source of mRNA expression by Lipid 16‐LNP, the tissue macrophages are a heterogeneous population depends on the expression of CD11b, CD11c, F4/80, and Ly6c. Hence, our future work should address details on macrophage subtype‐tropism.^[^ ^17^ ^]^ In conclusion, our study identified a novel, cell type‐specific lipid (Lipid 16) for macrophages and a superior lipid (Lipid 23) for liver targeting via vigorous in vitro and in vivo screening experiments and suggested that the combination of their structural modules could drive the cell specificity (Figure 5c). The intrinsic cell type‐specificity of amino ionizable based‐LNPs opens new avenues for future development of efficient mRNA therapeutics.

## Conflict of Interest

D.P. declares the following competing financial interest(s): D.P. receives licensing fees (or on scientific advisory boards or boards of directors) for, lectured (and received a fee), or conducts sponsored research at TAU for the following entities: ART Biosciences, BioNtech SE, Earli Inc., Kernal Biologics, Merck, Newphase Ltd., NeoVac Ltd., RiboX Therapeutics, Roche, SirTLabs Corporation, Teva Pharmaceuticals Inc. All other authors declare no competing interests.

## Supporting information

Supporting Information

## Data Availability

The data that support the findings of this study are available from the corresponding author upon reasonable request.
