# Supplementary material for: A Combinatorial Library of Lipid Nanoparticles for Cell Type‐Specific mRNA Delivery
Source: Adv Sci (Weinh). 2023 Apr 24;10(19):2301929. doi: 10.1002/advs.202301929 (PMC10323619; doi:10.1002/advs.202301929)
Supplement: Supplementary file 1 — Supporting Information [file ADVS-10-2301929-s001.pdf]

ADVANCED SCIENCE
Open Access

Supporting Information

for *Adv. Sci.*, DOI 10.1002/advs.202301929

A Combinatorial Library of Lipid Nanoparticles for Cell Type-Specific mRNA Delivery

*Gonna Somu Naidu, Seok-Beom Yong, Srinivas Ramishetti, Riccardo Rampado, Preeti Sharma, Assaf Ezra, Meir Goldsmith, Inbal Hazan-Halevy, Sushmita Chatterjee, Anjaiah Aitha and Dan Peer**

Supporting Information

A Combinatorial Library of Lipid Nanoparticles for Cell Type-Specific mRNA delivery

*Gonna Somu Naidu[1], Seok-Beom Yong[1], Srinivas Ramishetti, Riccardo Rampado, Preeti Sharma, Assaf Ezra, Meir Goldsmith, Inbal Hazan-Halevy, Sushmita Chatterjee, Anjaiah Aitha, Dan Peer**

Materials and Method.

Lipid synthesis: All reactions were performed in an oven-dried (120 °C) glass apparatus. All the chemicals were purchased from Sigma Aldrich unless mentioned. Linoleyl alcohol was obtained from TCI chemicals. Anhydrous dichloromethane (CH_2Cl_2) was purchased from Aldrich. Thin Layer Chromatography (TLC) was carried out using Merck silica gel 60 F_{254} plates. Column chromatography was performed on silica gel 60A (0.063-0.2 mm). ^{1}H NMR spectra were recorded on a 400 MHz spectrometer using $CDCl_3$ as the solvent, and the spectra were referenced to residual chloroform (δ 7.26 ppm). Mass spectra were recorded on an ESI-TOF mass spectrometer.

All starting aldehydes, **Lipid 6** and **Lipid 8**, were synthesized according to our previously reported procedures[1].

1. **Synthesis of Lipid 16**

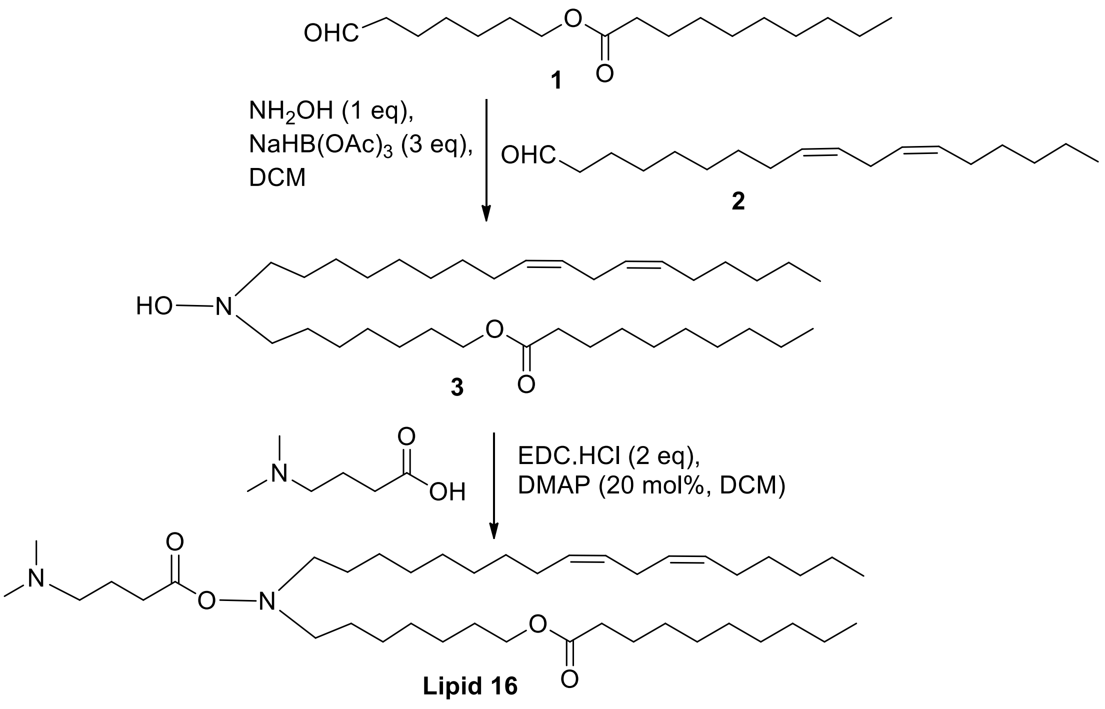

7-(Hydroxy((9Z,12Z)-octadeca-9,12-dien-1-yl)amino)heptyl decanoate

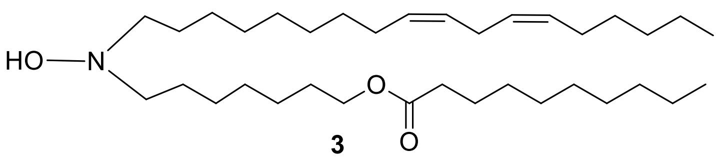

3

To a suspension of hydroxylamine hydrochloride (78 mg, 1.12 mmol, 1.0 equiv.) in dry CH_2Cl_2 (5 mL), triethylamine (156 µL, 1.12 mmol, 1.0 equiv.) was added under argon atmosphere and stirred for 5 min at room temperature. After that, a solution of 7-oxoheptyl decanoate **1** (319 mg, 1.12 mmol, 1.0 equiv.) in dry CH_2Cl_2 (15 mL) was added, drop wisely, and stirred for 2 hr. Later, sodium triacetoxyborohydride (354 mg, 1.68 mmol, 1.5 equiv.) was added portion-wise and stirred for 10 min. Then a solution of linoleyl aldehyde **2** (296 mg, 1.12 mmol, 1.0 equiv.) in dry CH_2Cl_2 (10 mL) was added, drop wisely, and stirred for another 10 min. Later the remaining amount of sodium triacetoxyborohydride (354 mg, 1.68 mmol, 1.5 equiv.) was added portion wise and stirred for 7 hr at room temperature under an argon atmosphere. The reaction was quenched with sat.$NaHCO_3$ solution and extracted with CH_2Cl_2 (3 times). The organic portion was washed with brine solution and dried over anhydrous Na_2SO_4. The solvent was evaporated, and the residue was purified by column chromatography using 0-10% ethyl acetate in hexane to obtain the desired hydroxylamine **3** (420 mg, 68%) as a pale yellow color liquid.

^{1}H NMR (400 MHz, $CDCl_3$): δ 5.44-5.26 (4 H, m), 5.04-5.91 (1 H, br) 4.05 (2 H, t, J = 6.8 Hz), 2.77 (2 H, t, J = 6.4 Hz), 2.62 (4 H, t, J = 7.6 Hz), 2.28 (2 H, t, J = 7.6 Hz), 2.05 (4 H, q, J = 6.8 Hz), 1.69-1.49 (8 H, m), 1.42-1.19 (34 H, m), 0.89 (3 H, t, J = 6.4 Hz), 0.88 (3 H, t, J = 6.8 Hz).

ESI-MS: *m/z* 550.70 [M+1]$^+$

7-(((4-(Dimethylamino)butanoyl)oxy)((9Z,12Z)-octadeca-9,12-dien-1-yl)amino)heptyl decanoate

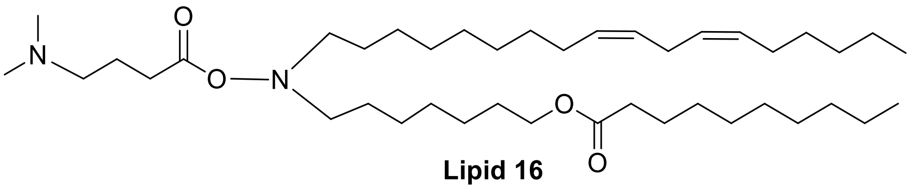

Lipid 16

The above hydroxylamine **3** (395 mg, 0.72 mmol, 1.0 equiv.), *N, N*-dimethyl aminobutyric acid hydrochloride (240 mg, 1.44 mmol, 2.0 equiv.), EDC.HCl (275 mg, 1.44 mmol, 2.0 equiv.) and DMAP (17 mg, 0.14 mmol, 0.2 equiv.) were dissolved in dry CH_2Cl_2 (20 mL) under argon atmosphere and stirred for 6 hr at room temperature. Then the reaction was quenched with sat. $NaHCO_3$ followed by extract with CH_2Cl_2 (3 times). The organic portion was washed with brine solution and dried over anhydrous Na_2SO_4. The solvent was evaporated, and the residue was purified by column chromatography using 0-6% IPA in $CHCl_3$ to obtain **Lipid 16** (296 mg, 63%) as pale yellow color oil.

^{1}H NMR (400 MHz, $CDCl_3$): δ 5.44-5.27 (4 H, m), 4.04 (2 H, t, J = 6.8 Hz), 2.79 (4 H, t, J = 7.4 Hz), 2.77 (2 H, t, J = 6.4 Hz), 2.32 (2 H, t, J = 7.6 Hz), 2.30 (2 H, t, J = 6.8 Hz), 2.28 (2 H, t, J = 7.6 Hz), 2.22 (6 H, s), 2.09-1.99 (4 H, m), 1.81 (2 H, quint, J = 7.2 Hz), 1.68-1.55 (4 H, m), 1.55-1.43 (4 H, m), 1.40-1.19 (34 H, m), 0.89 (3 H, t, J = 6.8 Hz), 0.88 (3 H, t, J = 7.2 Hz).

ESI-MS: m/z 663.84 $[M+1]^+$; 685.80 $[M+Na]^+$

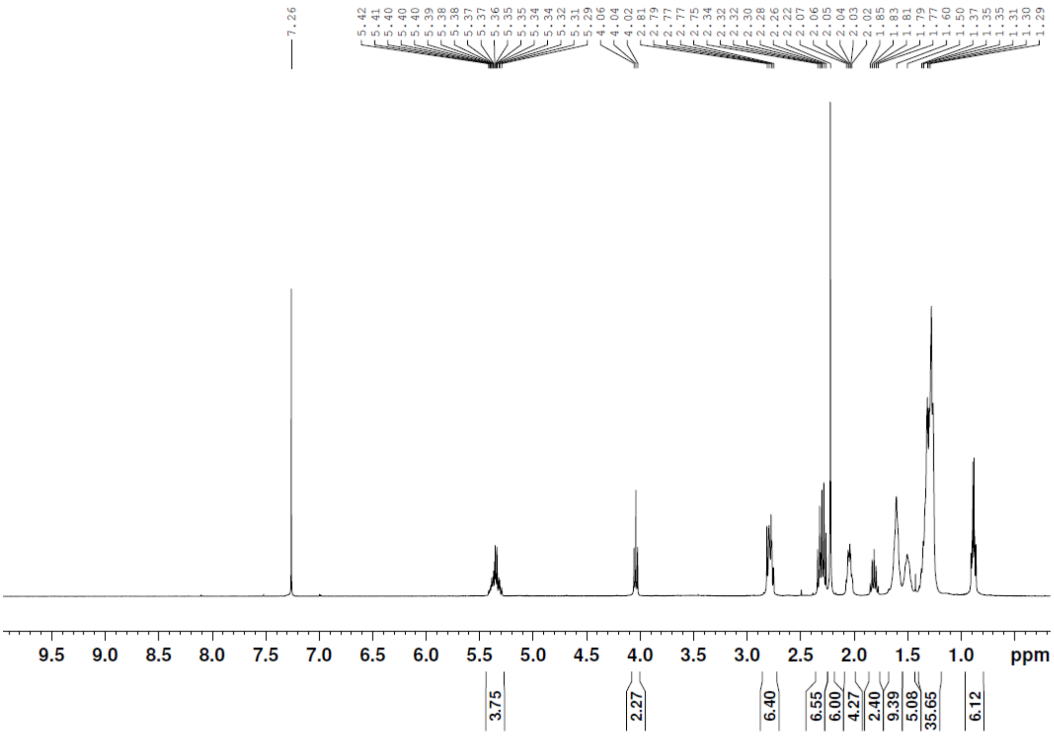

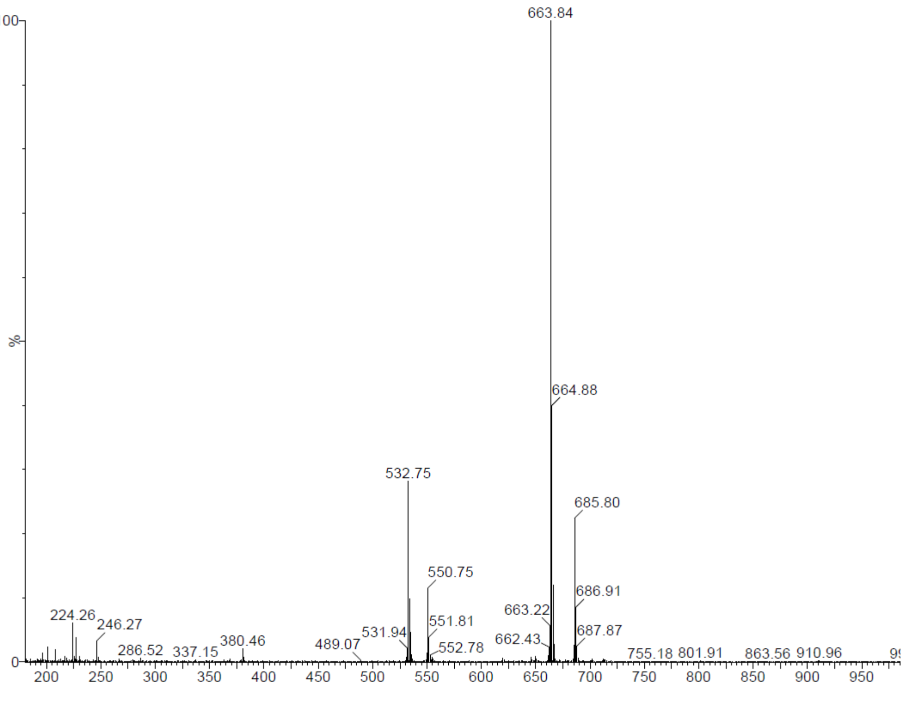

2. Synthesis of Lipid 17

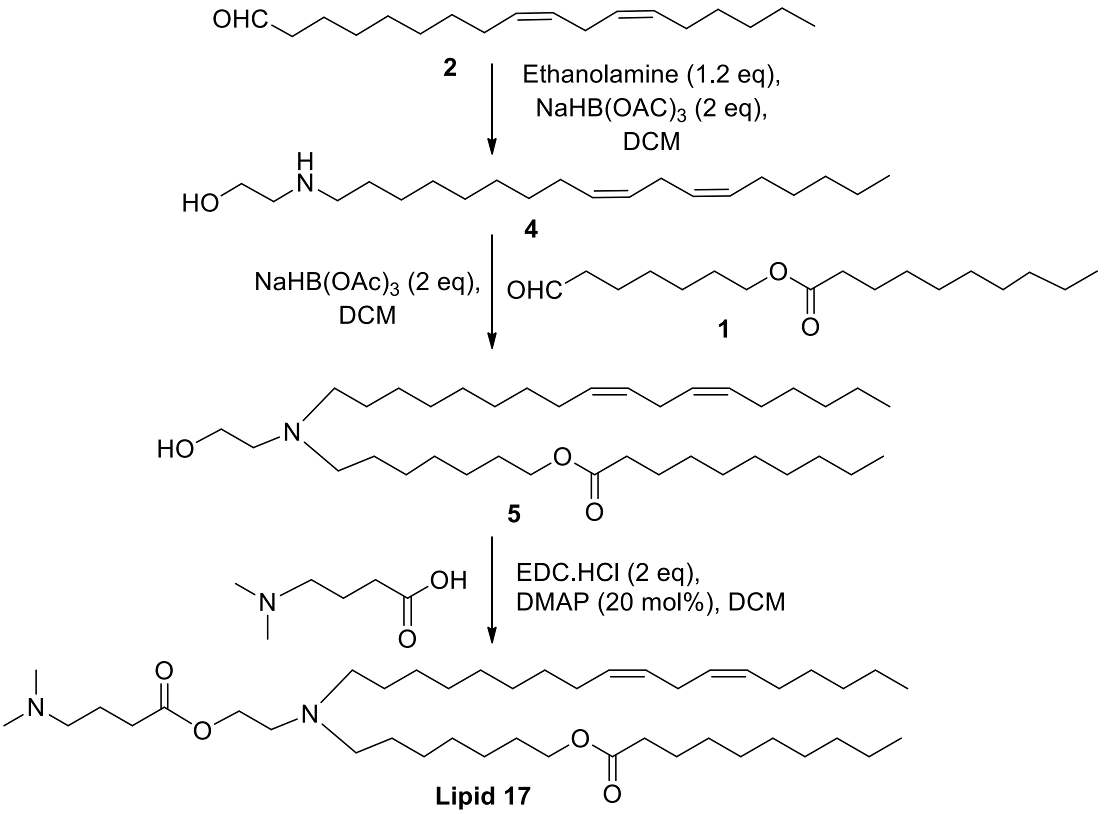

2-(((9Z, 12Z)-Octadeca-9, 12-dien-1-yl) amino) ethan-1-ol

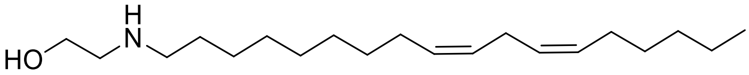

4

Linoleyl aldehyde **2** (3.2 g, 12.12 mmol, 1 equiv.) and ethanolamine (0.89 ml, 14.54 mmol, 1.2 equiv.) were dissolved in a dry CH_2Cl_2 (60 mL) under a nitrogen atmosphere and stirred for 1 hr at room temperature. Then sodium triacetoxyborohydride (5.1 g, 24.24 mmol, 2 equiv.) was added portion wise and stirred overnight at the same temperature. After that, the reaction was quenched with sat.$NaHCO_3$ solution followed by extract with CH_2Cl_2 (3 times). The organic layer was washed with brine solution and dried over anhydrous Na_2SO_4. The solvent was evaporated, and the residue was purified by column chromatography using 0-10% MeOH in $CHCl_3$ to obtain linoleyl ethanolamine **4** (2.0 g, 54%) as a pale yellowish liquid.

^{1}H NMR (400 MHz, $CDCl_3$): δ 5.40-5.30 (4 H, m), 3.64 (2 H, t, J = 5.2 Hz), 2.83-2.73 (4 H, m), 2.62 (2 H, t, J = 7.2 Hz), 2.04 (4 H, q, J = 6.8 Hz), 1.54-1.43 (2 H, m), 1.41-1.21 (16 H, m), 0.88 (3 H, t, J = 6.8 Hz).

ESI-MS: *m/z* 310.5 [M+1]$^+$

7-((2-Hydroxyethyl)((9Z,12Z)-octadeca-9,12-dien-1-yl)amino)heptyl decanoate

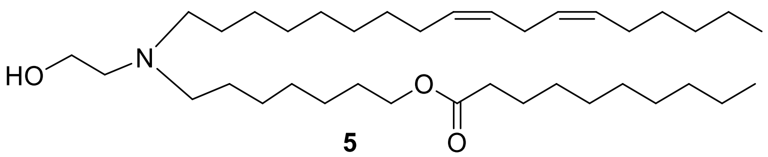

5

To linoleyl ethanolamine **4** (297 mg, 0.96 mmol, 1.0 equiv.), a solution of 7-oxoheptyl decanoate **1** (300 mg, 1.06 mmol, 1.1 equiv.) in dry CH_2Cl_2 (20 mL) was added under nitrogen atmosphere and stirred for 1 hr at room temperature. Then sodium triacetoxyborohydride (405 mg, 1.92 mmol, 2.0 equiv.) was added and stirred overnight at the same temperature. Later, the reaction was quenched with sat.$NaHCO_3$ solution followed by extract with CH_2Cl_2 (3 times). The organic layer was washed with brine solution and dried over anhydrous Na_2SO_4. The solvent was evaporated on a rotary evaporator, and the residue was purified by column chromatography using 0-2% MeOH in $CHCl_3$ to provide desired ethanolamine **5** (520 mg, 94%) as pale yellowish oil.

^{1}H NMR (400 MHz, $CDCl_3$): δ 5.43-5.27 (4 H, m), 4.05 (2 H, t, J = 6.8 Hz), 3.58 (2 H, t, J = 5.2 Hz), 2.77 (2 H, t, J = 6.4 Hz), 2.64 (2 H, t, J = 5.2 Hz), 2.52 (4 H, t, J = 7.6 Hz), 2.28 (2 H, t, J = 7.6 Hz), 2.04 (4 H, q, J = 6.8 Hz), 1.67-1.54 (4 H, m), 1.53-1.41 (4 H, m), 1.40-1.18 (34 H, m), 0.88 (3 H, t, J = 6.8 Hz), 0.87 (3 H, t, J = 6.8 Hz).

ESI-MS: *m/z* 578.8 [M+1]$^+$

7-((2-((4-(Dimethylamino)butanoyl)oxy)ethyl)((9Z,12Z)-octadeca-9,12-dien-1-yl)amino)heptyl decanoate

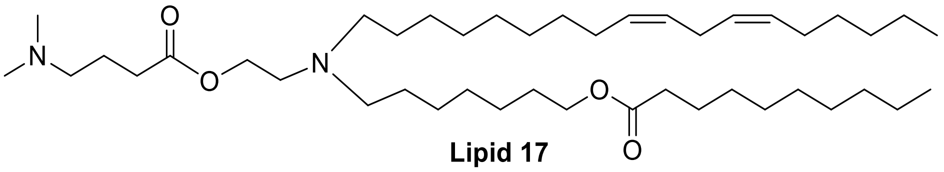

Lipid 17

The above ethanolamine **5** (500 mg, 0.87 mmol, 1.0 equiv.), *N, N*-dimethyl aminobutyric acid hydrochloride (289 mg, 1.73 mmol, 2.0 equiv.), EDC.HCl (331 mg, 1.73 mmol, 2.0 equiv.) and DMAP (10 mg, 0.087 mmol, 0.1 equiv.) were dissolved in dry CH_2Cl_2 (20 mL) under argon atmosphere and stirred for 24 hr at room temperature. After that, the reaction was quenched with sat. $NaHCO_3$ followed by extract with CH_2Cl_2 (3 times), washed with brine solution, and dried over with anhydrous Na_2SO_4. The solvent was evaporated, and the residue was purified

by column chromatography using 0-10% IPA in $CHCl_3$ to obtain **Lipid 17** (495 mg, 83%) as pale yellow color oil.

^{1}H NMR (400 MHz, $CDCl_3$): δ 5.43-5.27 (4 H, m), 4.11 (2 H, t, J = 6.4 Hz), 4.05 (2 H, t, J = 6.8 Hz), 2.77 (2 H, t, J = 6.4 Hz), 2.67 (2 H, t, J = 6.4 Hz), 2.44 (4 H, t, J = 7.6 Hz), 2.37-2.25 (6 H, m), 2.23 (6 H, s), 2.04 (4 H, q, J = 6.8 Hz), 1.79 (2 H, quint, J = 7.2 Hz), 1.67-1.54 (4 H, m), 1.47-1.17 (38 H, m), 0.89 (3 H, t, J = 6.8 Hz), 0.87 (3 H, t, J = 6.8 Hz).

ESI-MS: m/z 692.0 $[M+1]^+$; 346.5 $[M/2+1]^+$

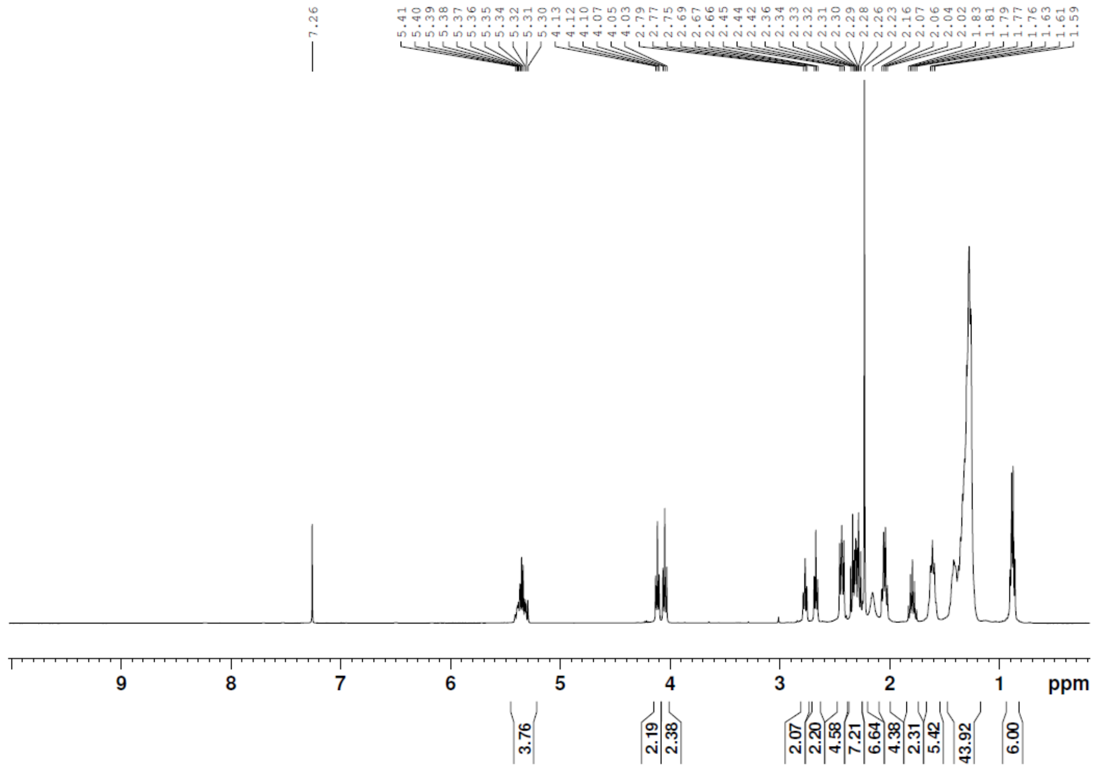

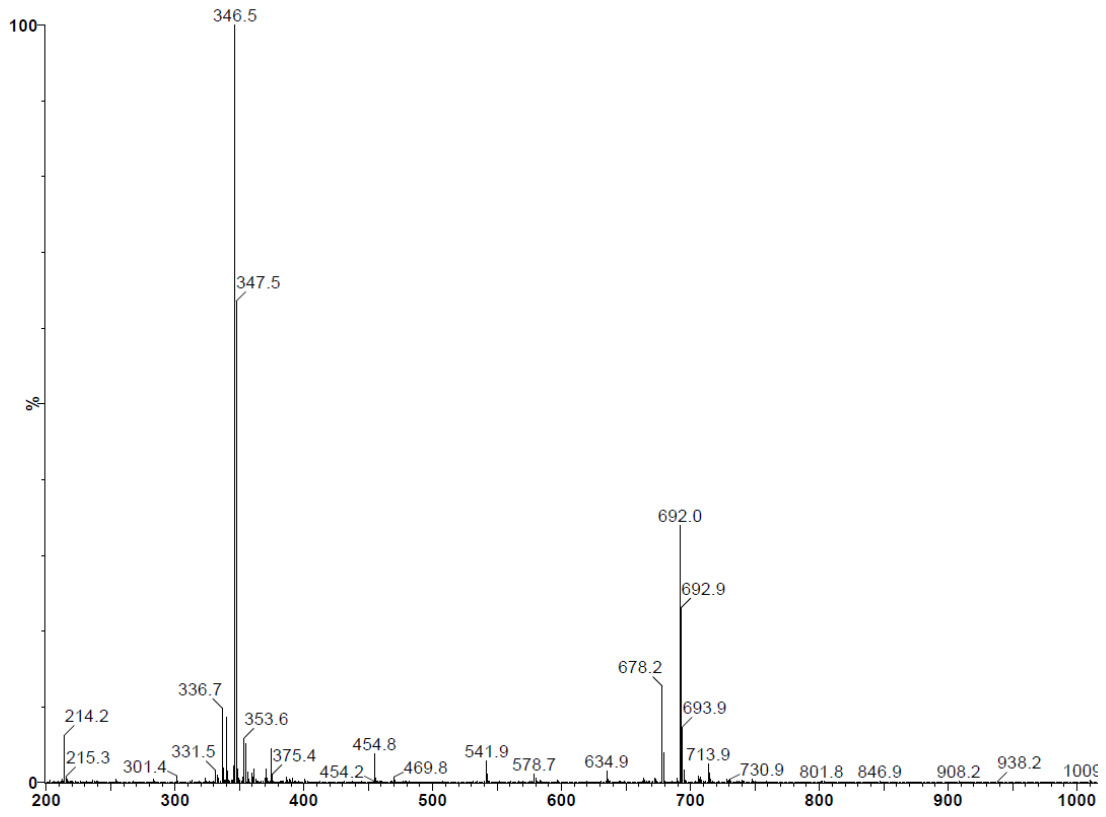

3. Synthesis of Lipid 18

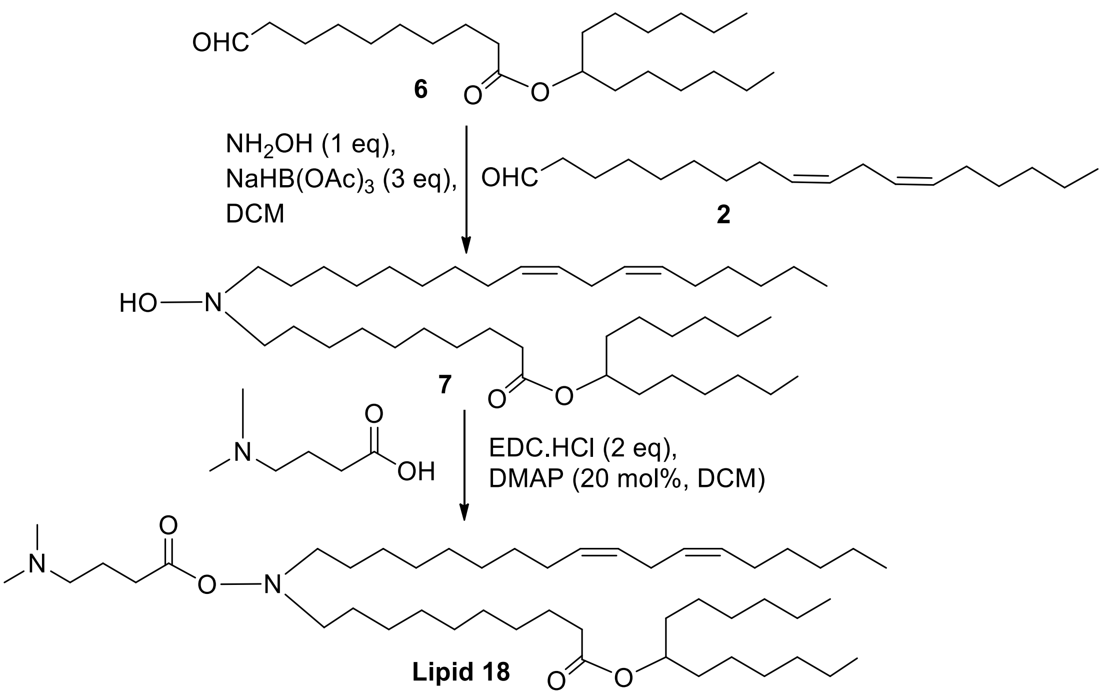

Tridecan-7-yl 10-(hydroxy((9Z,12Z)-octadeca-9,12-dien-1-yl)amino)decanoate

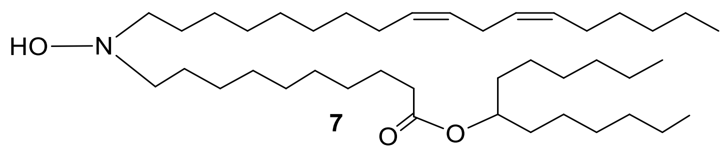

To a suspension of hydroxylamine hydrochloride (56 mg, 0.8 mmol, 1 equiv.) in dry CH_2Cl_2 (5 mL), dry triethylamine (112 μL, 0.80 mmol, 1 equiv.) was added under argon atmosphere, and stirred for 5 min at room temperature. Then, a solution of tridecan-7-yl 10-oxodecanoate **6** (295 mg, 0.80 mmol, 1.0 equiv.) in dry CH_2Cl_2 (15 mL) was added drop wisely and stirred for 2 hr. After that, sodium triacetoxyborohydride (250 mg, 1.20 mmol, 1.5 equiv.) was added portion wise and stirred for 10 min. Then a solution of linoleyl aldehyde **2** (232 mg, 0.88 mmol, 1.1 equiv.) in dry CH_2Cl_2 (10 mL) was added, drop wisely, and stirred for another 5 min. Later, the remaining amount of sodium triacetoxyborohydride (250 mg, 1.20 mmol, 1.5 equiv.) was added portion wise and stirred for 8 hr at room temperature under an argon atmosphere. The reaction was quenched with sat.$NaHCO_3$ solution and extracted with CH_2Cl_2 (3 times). The organic portion was washed with brine solution and dried over anhydrous Na_2SO_4. The solvent was evaporated, and the residue was purified by column chromatography using 0-10% ethyl acetate in hexane to obtain the desired hydroxylamine **7** (405 mg, 80%) as a colorless liquid.

^{1}H NMR (400 MHz, $CDCl_3$): δ 5.45-5.26 (4 H, m), 4.86 (1 H, quint, J = 6.0 Hz), 2.77 (2 H, t, J = 6.4 Hz), 2.61 (4 H, t, J = 7.6 Hz), 2.27 (2 H, t, J = 7.6 Hz), 2.04 (4 H, q, J = 6.8 Hz), 1.70-1.43 (12 H, m), 1.42-1.11 (40 H, m), 0.89 (3 H, t, J = 6.8 Hz), 0.87 (6 H, t, J = 6.8 Hz).

ESI-MS: *m/z* 634.9 $[M+1]^+$

Tridecan-7-yl 10-(((4-(dimethylamino)butanoyl)oxy)((9Z,12Z)-octadeca-9,12-dien-1-yl)amino)decanoate

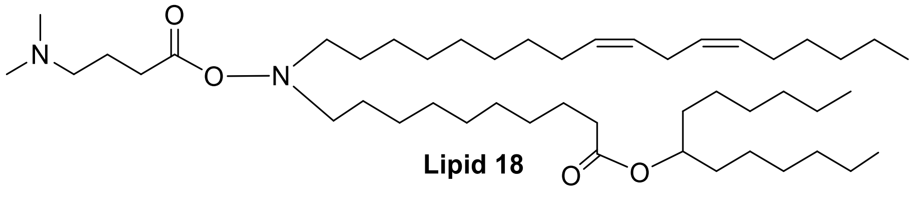

The above hydroxylamine **7** (400 mg, 0.63 mmol, 1.0 equiv.), 4-(dimethylamino)butyric acid hydrochloride (210 mg, 1.26 mmol, 2.0 equiv.), EDC.HCl (240 mg, 1.26 mmol, 2.0 equiv.) and DMAP (15 mg, 0.13 mmol, 20 mol%) were dissolved in dry CH_2Cl_2 (20 mL) under an argon atmosphere and stirred for 6 hr at room temperature. After that, the reaction was quenched with sat. $NaHCO_3$ followed by extract with CH_2Cl_2 (3 times). Then the organic portion was washed

with brine solution and dried over anhydrous Na_2SO_4. The solvent was evaporated, and the residue was purified by column chromatography using 0-10% IPA in $CHCl_3$ to bestow **Lipid 18** (367 mg, 78%) as a colorless oil.

^{1}H NMR (400 MHz, $CDCl_3$): δ 5.46-5.27 (4 H, m), 4.86 (1 H, quint, J = 6.0 Hz), 2.88-2.68 (6 H, m), 2.36-2.24 (6 H, m), 2.22 (6 H, s), 2.09-1.98 (4 H, m), 1.81 (2 H, quint, J = 7.2 Hz), 1.62-1.54 (4 H, m), 1.54-1.41 (8 H, m), 1.40-1.15 (40 H, m), 0.89 (3 H, t, J = 6.4 Hz), 0.87 (6 H, t, J = 6.8 Hz).

ESI-MS: m/z 748.1 $[M+1]^+$

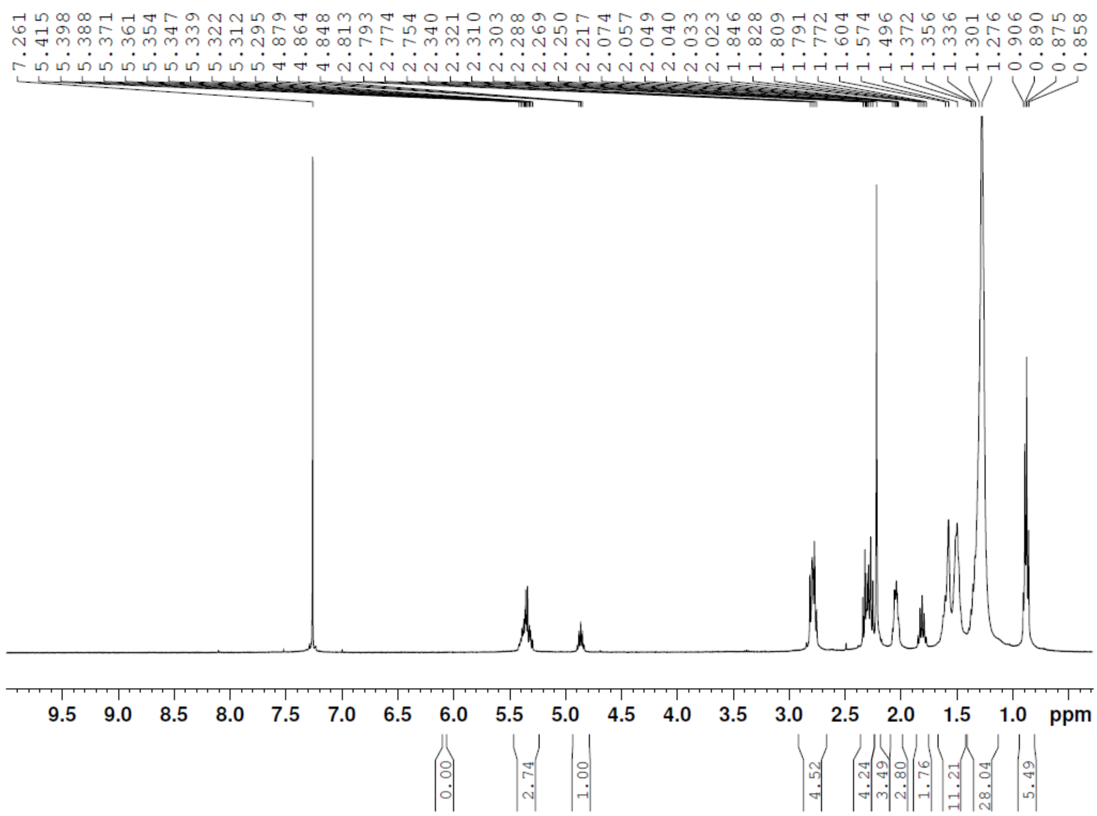

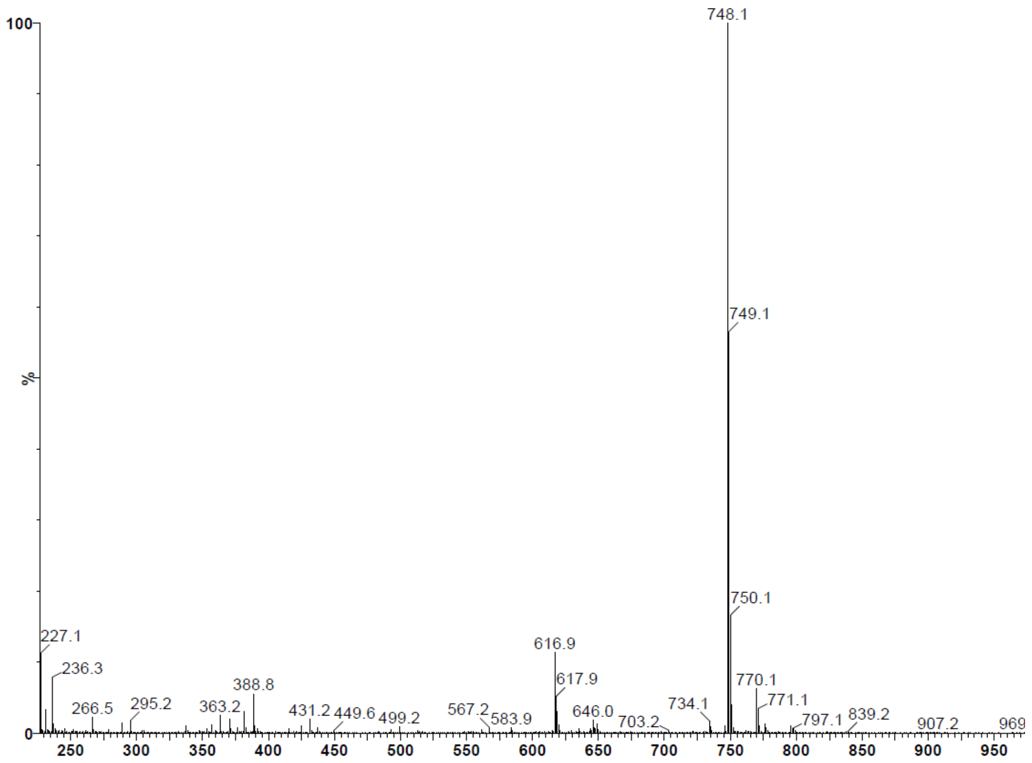

4. Synthesis of Lipid 19

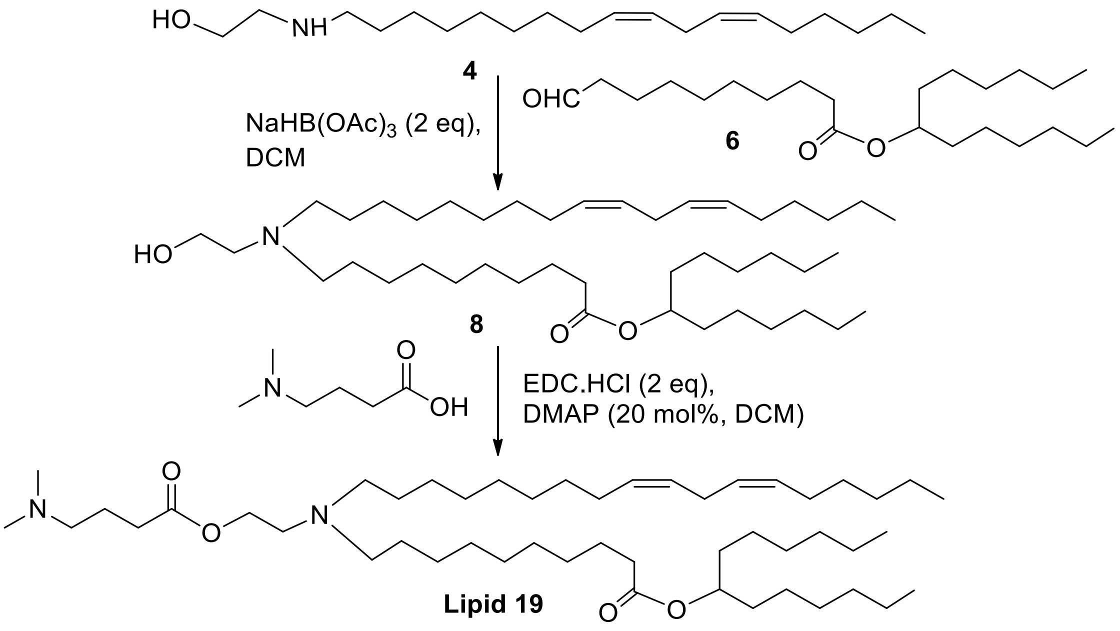

Tridecan-7-yl 10-((2-hydroxyethyl)((9Z,12Z)-octadeca-9,12-dien-1-yl)amino)decanoate

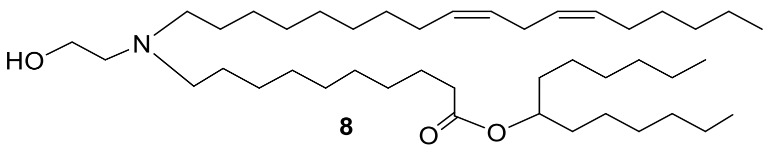

8

To the linoleyl ethanolamine **4** (232 mg, 0.75 mmol, 1.0 equiv.), a solution of tridecan-7-yl 10-oxodecanoate **6** (302 mg, 0.82 mmol, 1.1 equiv.) in dry CH_2Cl_2 (20 mL) was added in under argon atmosphere and stirred for 2 hr at room temperature. Then sodium triacetoxyborohydride (316 mg, 1.50 mmol, 2.0 equiv.) was added and stirred overnight at the same temperature. Later the reaction was quenched with sat.$NaHCO_3$ solution followed by extract with CH_2Cl_2 (3 times). The organic layer was washed with brine solution and dried over anhydrous Na_2SO_4. The solvent was evaporated on a rotary evaporator, and the residue was purified by column chromatography using 0-5% Isopropanol in $CHCl_3$ to obtain the desired ethanolamine **8** (417 mg, 84%) as a colorless liquid.

^{1}H NMR (400 MHz, $CDCl_3$): δ 5.45-5.25 (4 H, m), 4.86 (1 H, quint, J = 6.4 Hz), 3.55 (2 H, t, J = 4.8 Hz), 2.77 (2 H, t, J = 6.4 Hz), 2.60 (2 H, t, J = 5.2 Hz), 2.47 (4 H, t, J = 7.2 Hz), 2.27 (2 H, t, J = 7.6 Hz), 2.05 (4 H, q, J = 6.8 Hz), 1.65-1.56 (2 H, m), 1.56-1.39 (10 H, m), 1.39-1.08 (40 H, m), 0.89 (3 H, t, J = 6.4 Hz), 0.87 (6 H, t, J = 6.8 Hz).

ESI-MS: *m/z* 663.0 $[M+1]^+$

Tridecan-7-yl 10-((2-((4-(dimethylamino)butanoyl)oxy)ethyl)((9Z,12Z)-octadeca-9,12-dien-1-yl)amino)decanoate

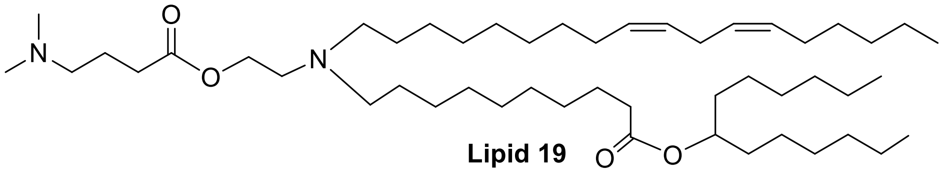

Lipid 19

The above ethanolamine **8** (415 mg, 0.63 mmol, 1.0 equiv.), *N, N*-dimethyl aminobutyric acid hydrochloride (209 mg, 1.25 mmol, 2.0 equiv.), EDC.HCl (239 mg, 1.25 mmol, 2.0 equiv.) and DMAP (15 mg, 0.12 mmol, 20 mol%) were dissolved in dry CH_2Cl_2 (20 mL) under argon atmosphere and stirred for 24 hr at room temperature. After that, the reaction was quenched with sat. $NaHCO_3$ followed by extract with CH_2Cl_2 (3 times). Then the organic portion was washed with brine solution and dried over anhydrous Na_2SO_4. The solvent was evaporated, and the residue was purified by column chromatography using 0-10% Isopropanol in $CHCl_3$ to obtain **Lipid 19** (365 mg, 75%) as a colorless liquid.

^{1}H NMR (400 MHz, $CDCl_3$): δ 5.45-5.27 (4 H, m), 4.87 (1 H, quint, J = 6.4 Hz), 4.12 (2 H, t, J = 6.4 Hz), 2.77 (2 H, t, J = 6.4 Hz), 2.67 (2 H, t, J = 6.4 Hz), 2.44 (4 H, t, J = 7.6 Hz), 2.34 (2 H, t, J = 7.6 Hz), 2.31-2.24 (4 H, m), 2.23 (6 H, s), 2.05 (4 H, q, J = 6.8 Hz), 1.79 (4 H, quint, J = 7.6 Hz), 1.61 (2 H, quint, J = 7.2 Hz), 1.55-1.46 (4 H, m), 1.45-1.08 (44 H, m), 0.89 (3 H,

t, J = 6.8 Hz), 0.87 (6 H, t, J = 6.8 Hz).

ESI-MS: m/z 776.1 $[M+1]^+$; 388.7 $[M/2+1]^+$

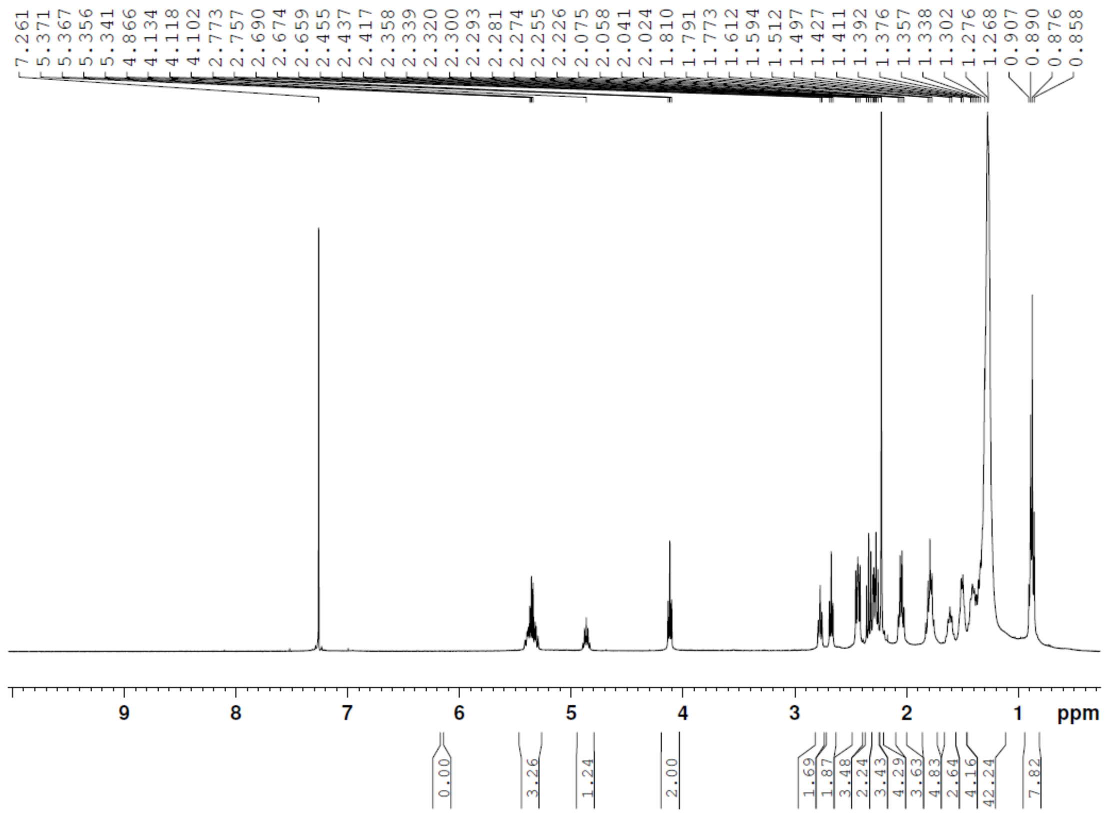

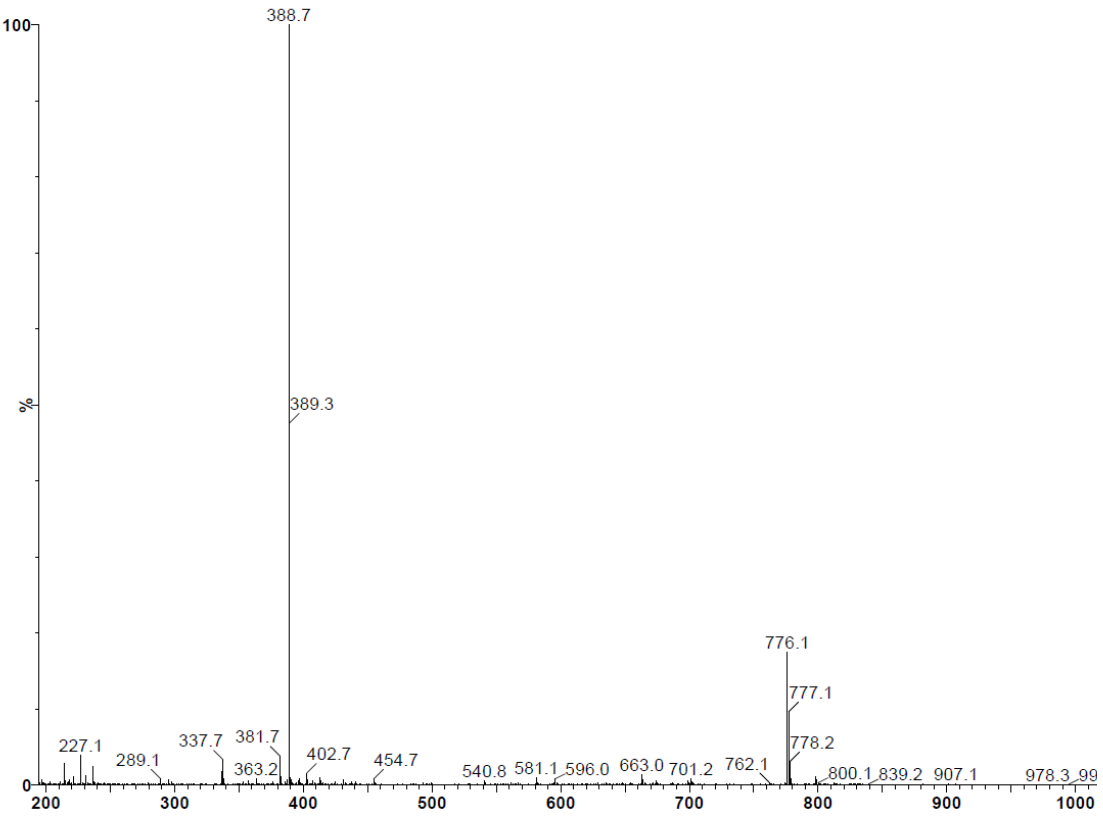

5. Synthesis of Lipid 20

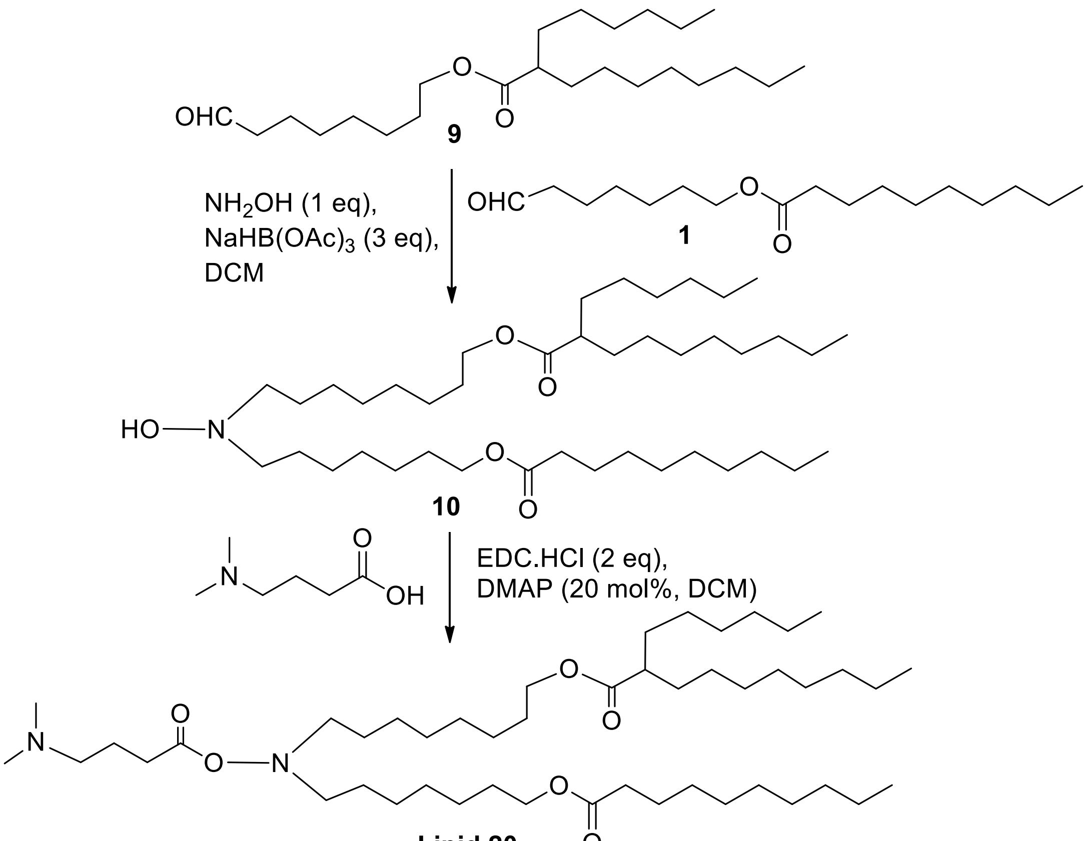

Lipid 20

8-((7-(Decanoyloxy)heptyl)(hydroxy)amino)octyl 2-hexyldecanoate

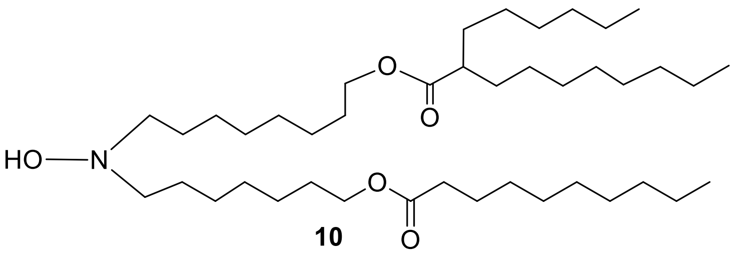

10

To a suspension of hydroxylamine hydrochloride (76 mg, 1.09 mmol, 1 equiv.) in dry CH_2Cl_2 (5 mL), dry triethylamine (152 µL, 1.09 mmol, 1 equiv.) was added under argon atmosphere, and stirred for 5 min at room temperature. Then, a solution of 8-oxooctyl 2-hexyldecanoate **9** (417 mg, 1.09 mmol, 1 equiv.) in dry CH_2Cl_2 (15 mL) was added drop wisely and stirred for 2 hr. After that, sodium triacetoxyborohydride (345 mg, 1.63 mmol, 1.5 equiv.) was added portion wise and stirred for 10 min. Then, a solution of 7-oxoheptyl decanoate **1** (310 mg, 1.09 mmol, 1 equiv.) in dry CH_2Cl_2 (10 mL) was added, drop wisely, and stirred for another 5 min. Later the remaining amount of sodium triacetoxyborohydride (345 mg, 1.63 mmol, 1.5 equiv.) was added portion wise and stirred for 9 hr at room temperature under an argon atmosphere. The

reaction was quenched with sat.$NaHCO_3$ solution and extracted with CH_2Cl_2 (3 times). The organic portion was washed with brine solution and dried over anhydrous Na_2SO_4. The solvent was evaporated, and the residue was purified by column chromatography using 0-10% ethyl acetate in hexane to obtain the desired hydroxylamine **10** (592 mg, 81%) as a colorless liquid.

^{1}H NMR (400 MHz, $CDCl_3$): δ 4.06 (2 H, t, J = 6.8 Hz), 4.05 (2 H, t, J = 6.8 Hz), 2.61 (4 H, t, J = 7.6 Hz), 2.36-2.24 (1 H, m), 2.28 (2 H, t, J = 7.6 Hz), 1.70- 1.50 (12 H, m), 1.48-1.13 (48 H, m), 0.92-0.82 (9 H, m).

ESI-MS: *m/z* 668.9 $[M+1]^+$

8-((7-(Decanoyloxy)heptyl)((4-(dimethylamino)butanoyl)oxy)amino)octyl 2-hexyldecanoate

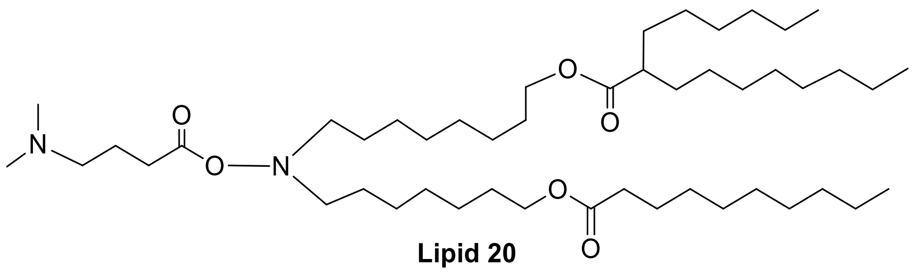

Lipid 20

The above hydroxylamine **10** (590 mg, 0.88 mmol, 1 equiv.), 4-(dimethylamino)butyric acid hydrochloride (295 mg, 1.77 mmol, 2 equiv.), EDC.HCl (337 mg, 1.77 mmol, 2 equiv.) and DMAP (22 mg, 0.18 mmol, 20 mol%) were dissolved in dry CH_2Cl_2 (20 mL) under argon atmosphere and stirred for 6 hr at room temperature. After that, the reaction was quenched with sat. $NaHCO_3$ followed by extract with CH_2Cl_2 (3 times). Then the organic portion was washed with brine solution and dried over with anhydrous Na_2SO_4. The solvent was evaporated, and the residue was purified by column chromatography using 0-6% IPA in $CHCl_3$ to bestow **Lipid 20** (473 mg, 69%) as a colorless liquid.

^{1}H NMR (400 MHz, $CDCl_3$): δ 4.05 (2 H, t, J = 6.8 Hz), 4.04 (2 H, t, J = 6.8 Hz), 2.79 (4 H, t, J = 7.6 Hz), 2.35-2.25 (1 H, m), 2.32 (2 H, t, J = 7.6 Hz), 2.29 (2 H, t, J = 7.2 Hz), 2.28 (2 H, t, J = 7.6 Hz), 2.21 (6 H, s), 1.81 (2 H, quint, J = 7.6 Hz), 1.71- 1.54 (8 H, m), 1.54-1.39 (6 H, m), 1.39-1.12 (44 H, m), 0.92-0.80 (9 H, m).

ESI-MS: *m/z* 782.0 $[M+1]^+$

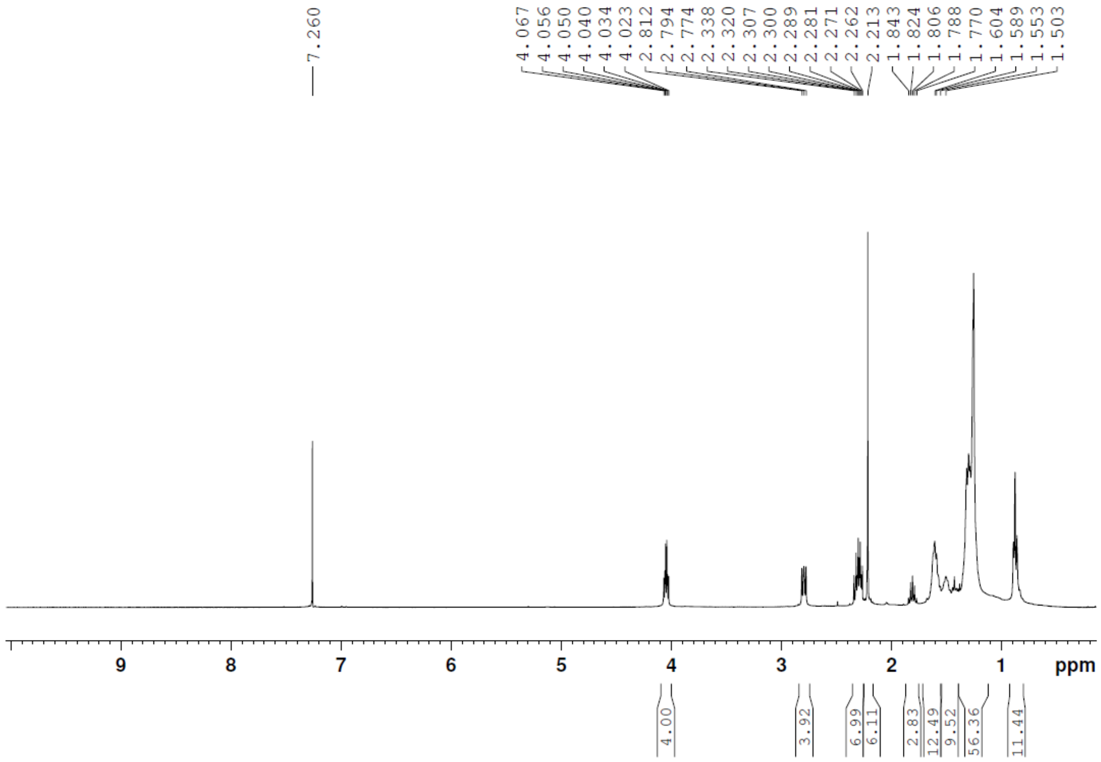

7.260
4.067
4.056
4.050
4.040
4.034
4.023
2.812
2.794
2.774
2.338
2.320
2.307
2.300
2.289
2.281
2.271
2.262
2.213
1.843
1.824
1.806
1.788
1.770
1.604
1.589
1.553
1.503
9
8
7
6
5
4
3
2
1
ppm
4.00
3.92
6.99
6.11
2.83
12.49
9.52
56.36
11.44

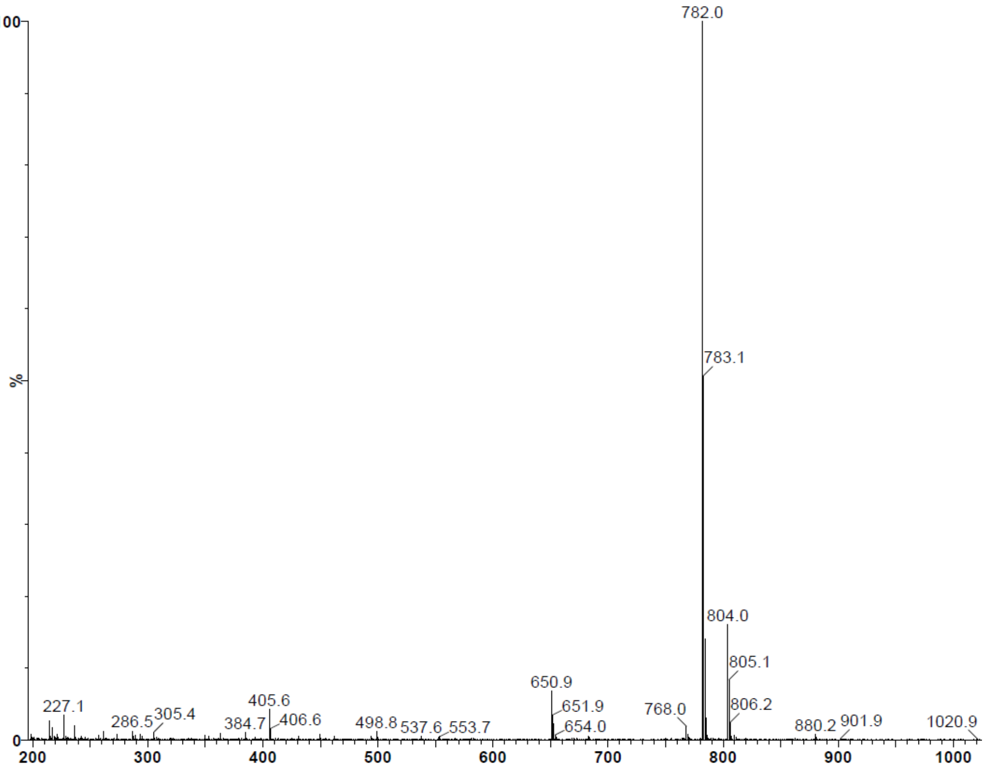

100
%
0
782.0
783.1
804.0
805.1
806.2
650.9
651.9
654.0
768.0
227.1
286.5
305.4
384.7
405.6
406.6
498.8
537.6
553.7
880.2
901.9
1020.9
200
300
400
500
600
700
800
900
1000

6. Synthesis of Lipid 21

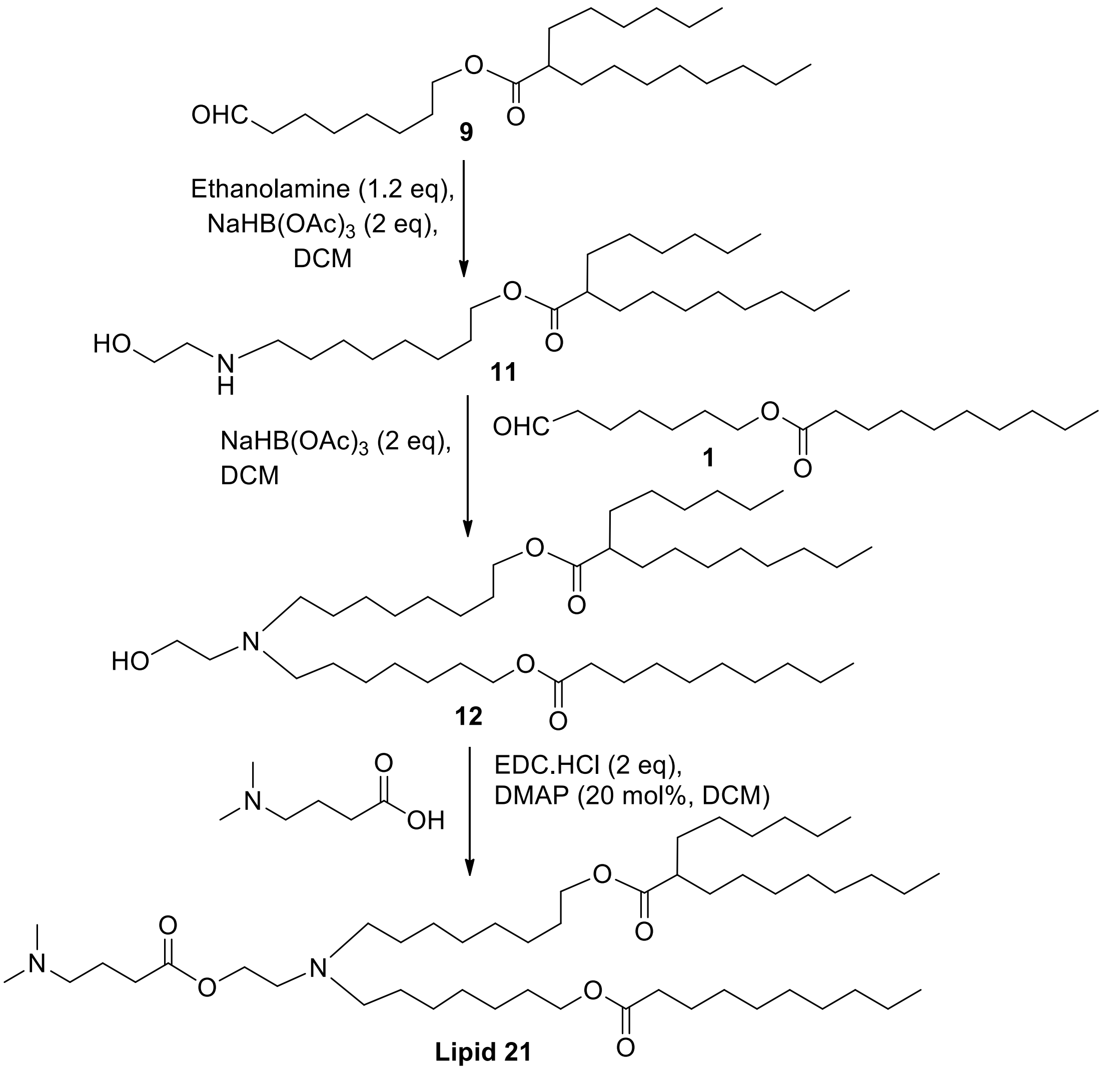

8-((2-hydroxyethyl)amino)octyl 2-hexyldecanoate

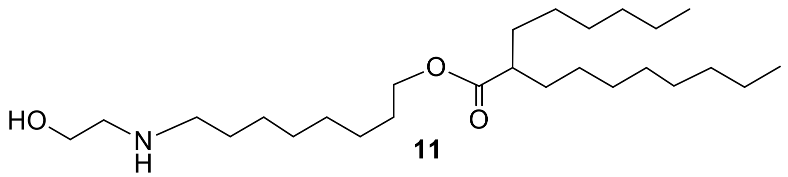

To a solution of 8-oxooctyl 2-hexyldecanoate **9** (910 mg, 2.38 mmol, 1 equiv.) in dry CH_2Cl_2 (80 mL), ethanolamine (174 µl, 2.86 mmol, 1.2 equiv.) was added under argon atmosphere and stirred for 2 hr at room temperature. Then sodium triacetoxyborohydride (1.0 g, 4.76 mmol, 2 equiv.) was added portion wise and stirred overnight at the same temperature. After that, the reaction was quenched with sat.$NaHCO_3$ solution followed by extract with CH_2Cl_2 (3 times). The organic layer was washed with brine solution and dried over anhydrous Na_2SO_4. The solvent was evaporated, and the residue was purified by column chromatography using 0-10% MeOH in $CHCl_3$ to get ethanolamine **11** (450 mg, 45 %) as a colorless liquid.

^1H NMR (400 MHz, $CDCl_3$): δ 4.06 (2 H, t, J = 6.8 Hz), 3.77 (2 H, t, J = 5.2 Hz), 2.92 (2 H, t, J = 5.2 Hz), 2.76 (2 H, t, J = 7.6 Hz), 2.36-2.25 (1 H, m), 1.71- 1.51 (6 H, m), 1.50-1.09 (30 H, m), 0.87 (6 H, t, J = 6.4 Hz).

ESI-MS: *m/z* 428.7 $[M+1]^+$

8-((7-(Decanoyloxy)heptyl)(2-hydroxyethyl)amino)octyl 2-hexyldecanoate

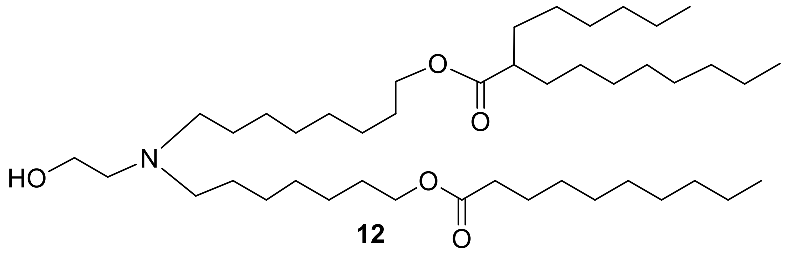

12

To the above ethanolamine, **11** (225 mg, 0.53 mmol, 1 equiv.), a solution of 7-oxoheptyl decanoate **1** (180 mg, 0.63 mmol, 1.2 equiv.) in dry CH_2Cl_2 (15 mL) was added in under argon atmosphere and stirred for 2 hr at room temperature. Later sodium triacetoxyborohydride (222 mg, 1.05 mmol, 2.0 equiv.) was added and stirred overnight at the same temperature. Then the reaction was quenched with sat.$NaHCO_3$ solution followed by extract with CH_2Cl_2 (3 times). The organic layer was washed with brine solution and dried over anhydrous Na_2SO_4. The solvent was evaporated on a rotary evaporator, and the residue was purified by column chromatography using 0-5% Isopropanol in $CHCl_3$ to obtain the desired ethanolamine **12** (297 mg, 81%) as colorless liquid.

^1H NMR (400 MHz, $CDCl_3$): δ 4.06 (2 H, t, J = 6.8 Hz), 4.05 (2 H, t, J = 6.8 Hz), 3.58 (2 H, t, J = 5.2 Hz), 2.64 (2 H, t, J = 5.2 Hz), 2.52 (4 H, t, J = 7.6 Hz), 2.35-2.23 (1 H, m), 2.29 (2 H, t, J = 7.6 Hz), 1.70-1.52 (8 H, m), 1.52-1.39 (6 H, m), 1.39-1.06 (46 H, m), 0.94-0.78 (9 H, m).

ESI-MS: *m/z* 697.0 $[M+1]^+$

8-((7-(Decanoyloxy)heptyl)(2-((4-(dimethylamino)butanoyl)oxy)ethyl)amino)octyl 2-hexyldecanoate

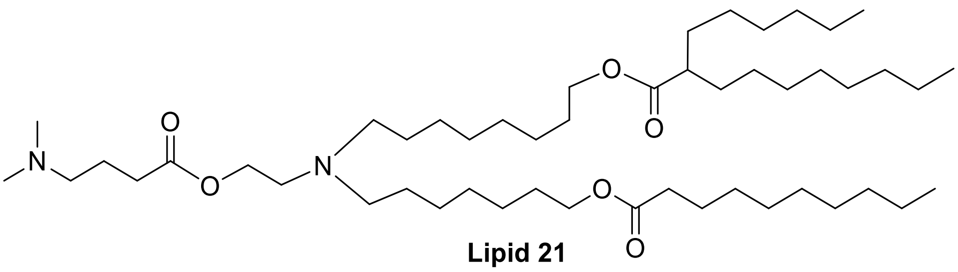

Lipid 21

The above ethanolamine **12** (295 mg, 0.42 mmol, 1 equiv.), *N, N*-dimethyl aminobutyric acid hydrochloride (141 mg, 0.85 mmol, 2 equiv.), EDC.HCl (162 mg, 0.85 mmol, 2 equiv.) and DMAP (10 mg, 0.08 mmol, 20 mol%) were dissolved in dry CH_2Cl_2 (20 mL) under an argon atmosphere and stirred overnight at room temperature. After that, the reaction was quenched with sat. $NaHCO_3$ followed by extract with CH_2Cl_2 (3 times). Then the organic portion was washed with brine solution and dried over with anhydrous Na_2SO_4. The solvent was evaporated, and the residue was purified by column chromatography using 0-10% Isopropanol in $CHCl_3$ to obtain **Lipid 21** (260 mg, 76%) as a pale yellowish liquid.

^{1}H NMR (400 MHz, $CDCl_3$): δ 4.12 (2 H, t, J = 6.4 Hz), 4.06 (2 H, t, J = 6.8 Hz), 4.05 (2 H, t, J = 6.8 Hz), 2.67 (2 H, t, J = 6.4 Hz), 2.44 (4 H, t, J = 7.6 Hz), 2.37-2.26 (1 H, m), 2.34 (2 H, t, J = 7.6 Hz), 2.32 (2 H, t, J = 6.8 Hz), 2.28 (2 H, t, J = 7.6 Hz), 2.24 (6 H, s), 1.80 (2 H, quint, J = 7.6 Hz), 1.69- 1.50 (10 H, m), 1.48-1.37 (6 H, m), 1.37-1.14 (44 H, m), 0.93-0.81 (9 H, m).

ESI-MS: *m/z* 810.1 $[M+1]^+$; 405.8 $[M/2+1]^+$

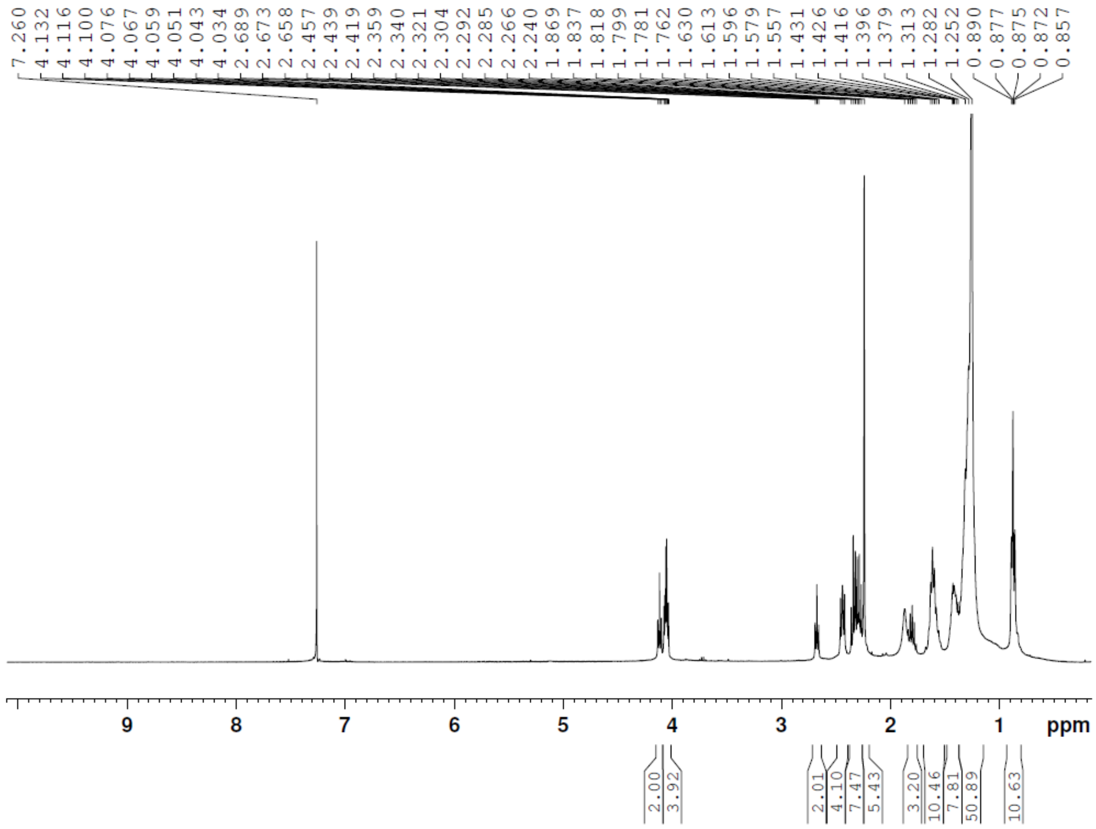
7.260
4.132
4.116
4.100
4.076
4.067
4.059
4.051
4.043
4.034
2.689
2.673
2.658
2.457
2.439
2.419
2.359
2.340
2.321
2.304
2.292
2.285
2.266
2.240
1.869
1.837
1.818
1.799
1.781
1.762
1.630
1.613
1.596
1.579
1.557
1.431
1.426
1.416
1.396
1.379
1.313
1.282
1.252
0.890
0.877
0.875
0.872
0.857
9
8
7
6
5
4
3
2
1
ppm
2.00
3.92
2.01
4.10
7.47
5.43
3.20
10.46
7.81
50.89
10.63

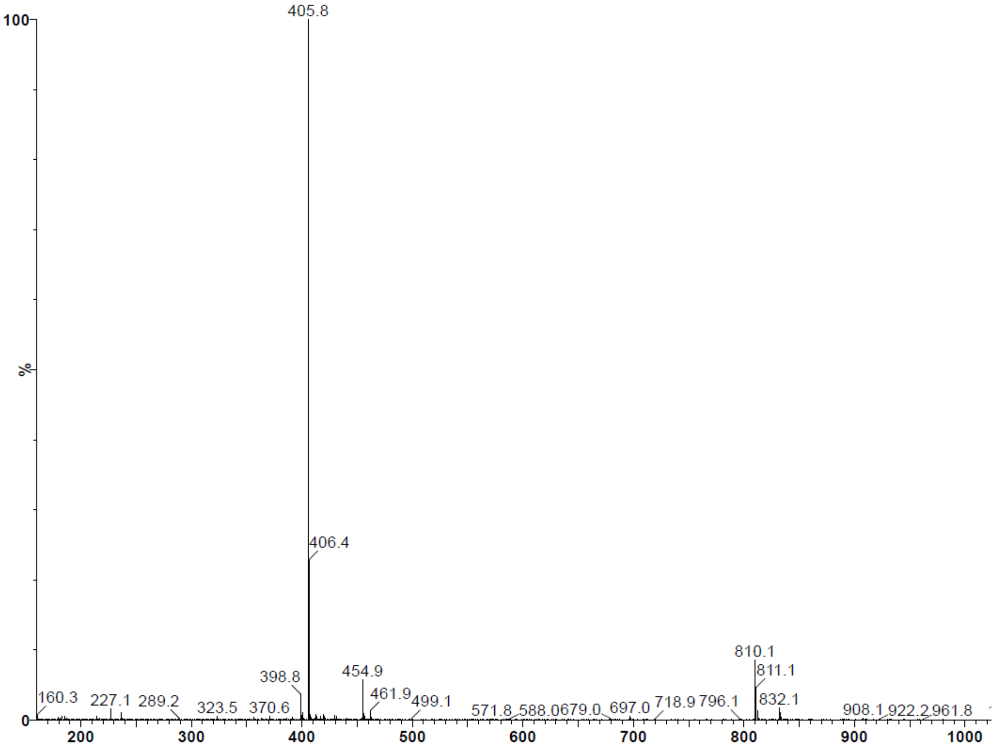
100
%
0
405.8
406.4
398.8
454.9
461.9
499.1
160.3
227.1
289.2
323.5
370.6
571.8
588.0
679.0
697.0
718.9
796.1
810.1
811.1
832.1
908.1
922.2
961.8
200
300
400
500
600
700
800
900
1000

7. Synthesis of Lipid 22

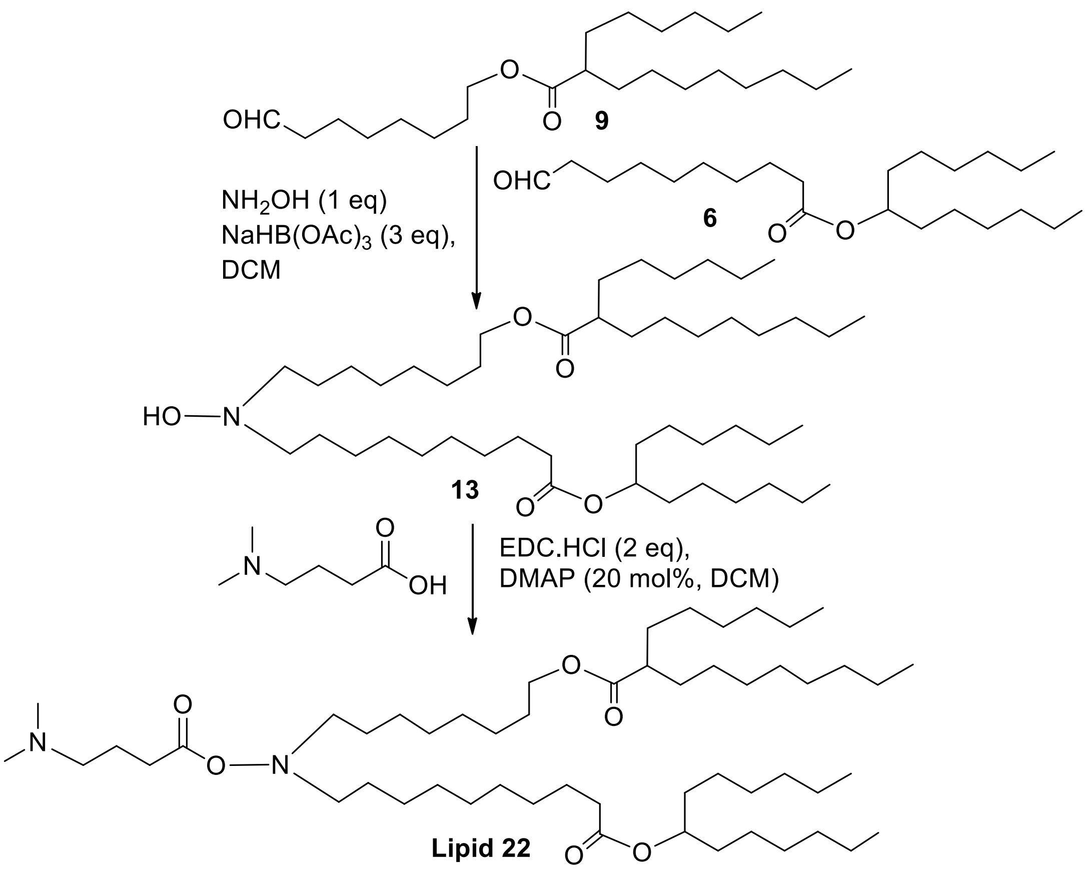

8-(Hydroxy(10-oxo-10-(tridecan-7-yloxy)decyl)amino)octyl 2-hexyldecanoate

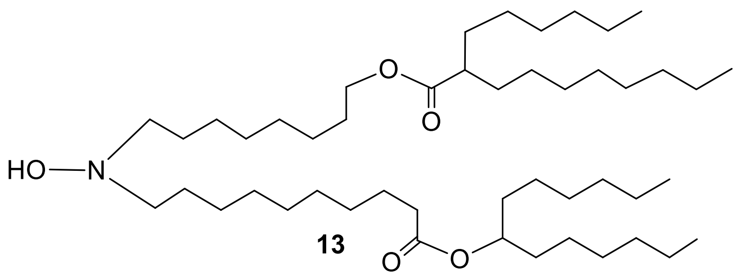

To a suspension of hydroxylamine hydrochloride (56 mg, 0.80 mmol, 1 equiv.) in dry CH_2Cl_2 (5 mL), dry triethylamine (111 μL, 0.80 mmol, 1 equiv.) was added under argon atmosphere, and stirred for 5 min at room temperature. Then, a solution of 8-oxooctyl 2-hexyldecanoate **9** (305 mg, 0.8 mmol, 1 equiv.) in dry CH_2Cl_2 (15 mL) was added drop wisely and stirred for 2 hr. After that, sodium triacetoxyborohydride (253 mg, 1.20 mmol, 1.5 equiv.) was added portion wise and stirred for 10 min. Then, a solution of tridecan-7-yl 10-oxodecanoate **6** (293 mg, 0.80 mmol, 1 equiv.) in dry CH_2Cl_2 (10 mL) was added drop wisely and stirred for another 5 min. Later, the remaining amount of sodium triacetoxyborohydride (253 mg, 1.2 mmol, 1.5 equiv.) was added portion wise and stirred for 8 hr at room temperature under an argon atmosphere. The reaction was quenched with sat.$NaHCO_3$ solution and extracted with CH_2Cl_2 (3 times). The

organic portion was washed with brine solution and dried over anhydrous Na_2SO_4. The solvent was evaporated, and the residue was purified by column chromatography using 0-10% ethyl acetate in hexane to obtain the desired hydroxylamine **13** (460 mg, 76%) as a colorless liquid.

^{1}H NMR (400 MHz, $CDCl_3$): δ 4.86 (1 H, quint, J = 6.0 Hz), 4.06 (2 H, t, J = 6.8 Hz), 2.61 (4 H, t, J = 7.6 Hz), 2.35-2.23 (1 H, m), 2.27 (2 H, t, J = 7.6 Hz), 1.70-1.39 (20 H, m), 1.39-1.10 (50 H, m), 0.87 (12 H, t, J = 7.2 Hz).

ESI-MS: *m/z* 753.0 $[M+1]^+$

8-(((4-(dimethylamino)butanoyl)oxy)(10-oxo-10-(tridecan-7-yloxy)decyl)amino)octyl 2-hexyldecanoate

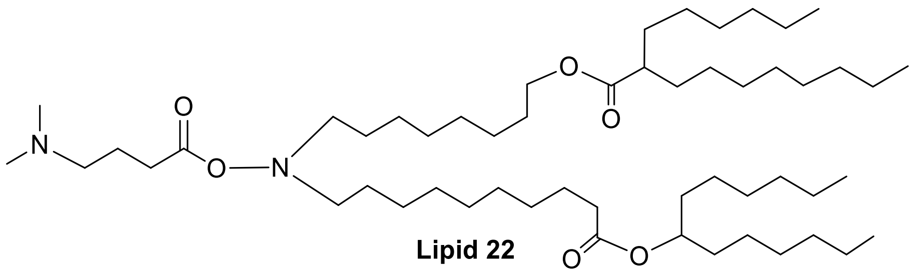

Lipid 22

The above hydroxylamine **13** (405 mg, 0.54 mmol, 1 equiv.), 4-(dimethylamino)butyric acid hydrochloride (180 mg, 1.08 mmol, 2 equiv.), EDC.HCl (206 mg, 1.08 mmol, 2 equiv.) and DMAP (13 mg, 0.11 mmol, 20 mol%) were dissolved in dry CH_2Cl_2 (20 mL) under argon atmosphere and stirred for 6 hr at room temperature. After that, the reaction was quenched with sat. $NaHCO_3$ followed by extract with CH_2Cl_2 (3 times). Then the organic portion was washed with brine solution and dried over anhydrous Na_2SO_4. The solvent was evaporated, and the residue was purified by column chromatography using 0-6% IPA in $CHCl_3$ to get **Lipid 22** (350 mg, 75%) as a colorless oil.

^{1}H NMR (400 MHz, $CDCl_3$): δ 4.86 (1 H, quint, J = 6.4 Hz), 4.05 (2 H, t, J = 6.8 Hz), 2.79 (4 H, t, J = 7.6 Hz), 2.36-2.23 (1 H, m), 2.32 (2 H, t, J = 7.6 Hz), 2.30 (2 H, t, J = 7.6 Hz), 2.27 (2 H, t, J = 7.6 Hz), 2.22 (6 H, s), 1.81 (2 H, quint, J = 7.6 Hz), 1.64-1.54 (6 H, m), 1.54-1.37 (10 H, m), 1.37-1.11 (54 H, m), 0.87 (12 H, t, J = 6.8 Hz).

ESI-MS: *m/z* 866.0 $[M+1]^+$

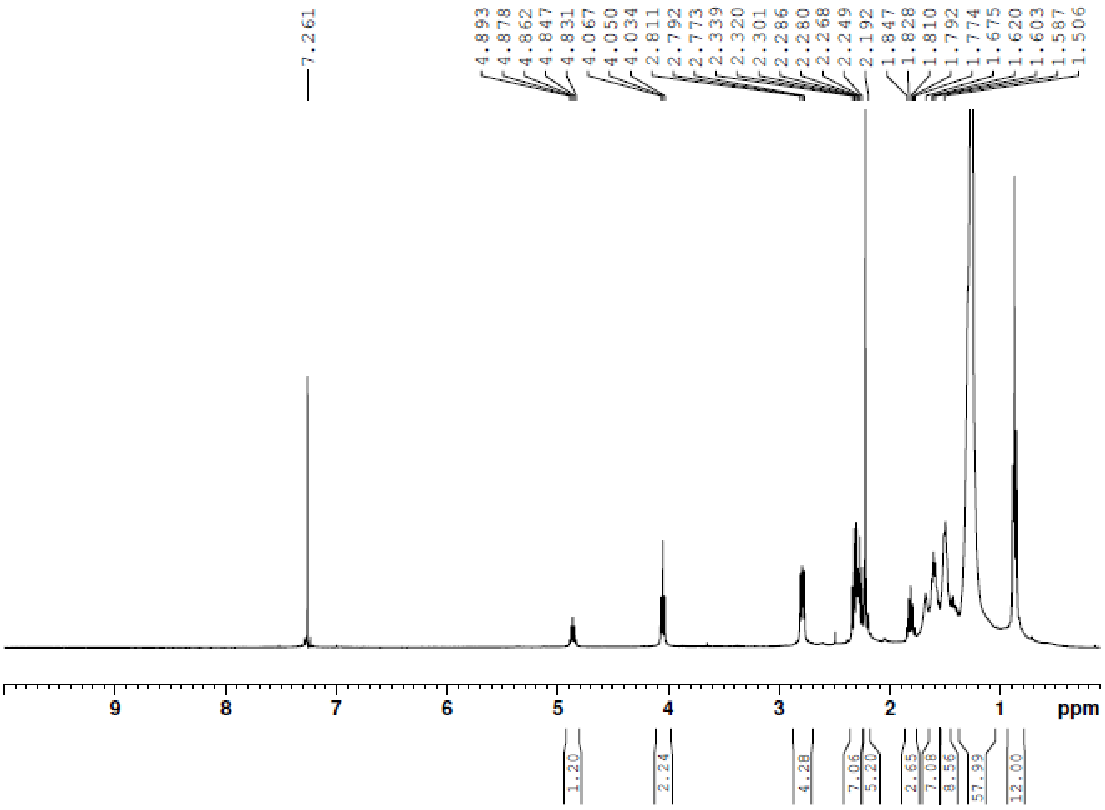

7.261
4.893
4.878
4.862
4.847
4.831
4.067
4.050
4.034
2.811
2.792
2.773
2.339
2.320
2.301
2.286
2.280
2.268
2.249
2.192
1.847
1.828
1.810
1.792
1.774
1.675
1.620
1.603
1.587
1.506
9
8
7
6
5
4
3
2
1
ppm
1.20
2.24
4.28
7.06
5.20
2.65
7.08
8.56
57.99
12.00

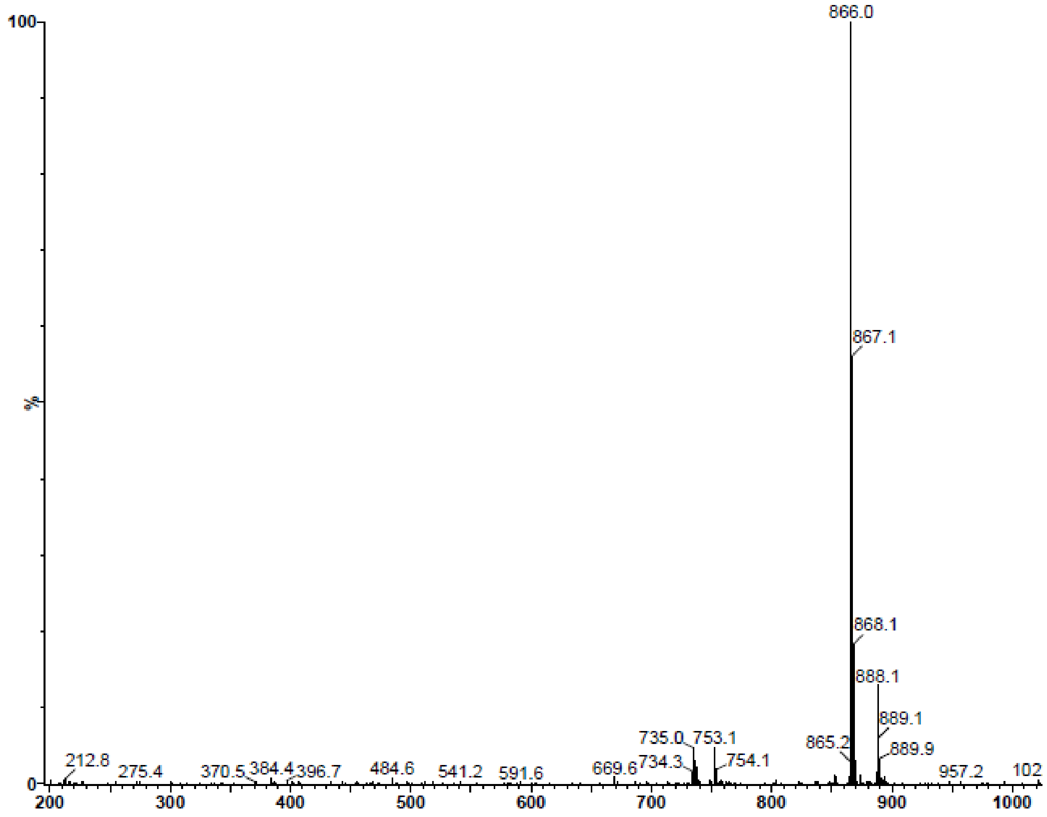

100
%
0
866.0
867.1
868.1
888.1
889.1
889.9
865.2
735.0
753.1
734.3
754.1
669.6
212.8
275.4
370.5
384.4
396.7
484.6
541.2
591.6
957.2
102
200
300
400
500
600
700
800
900
1000

8. Synthesis of Lipid 23

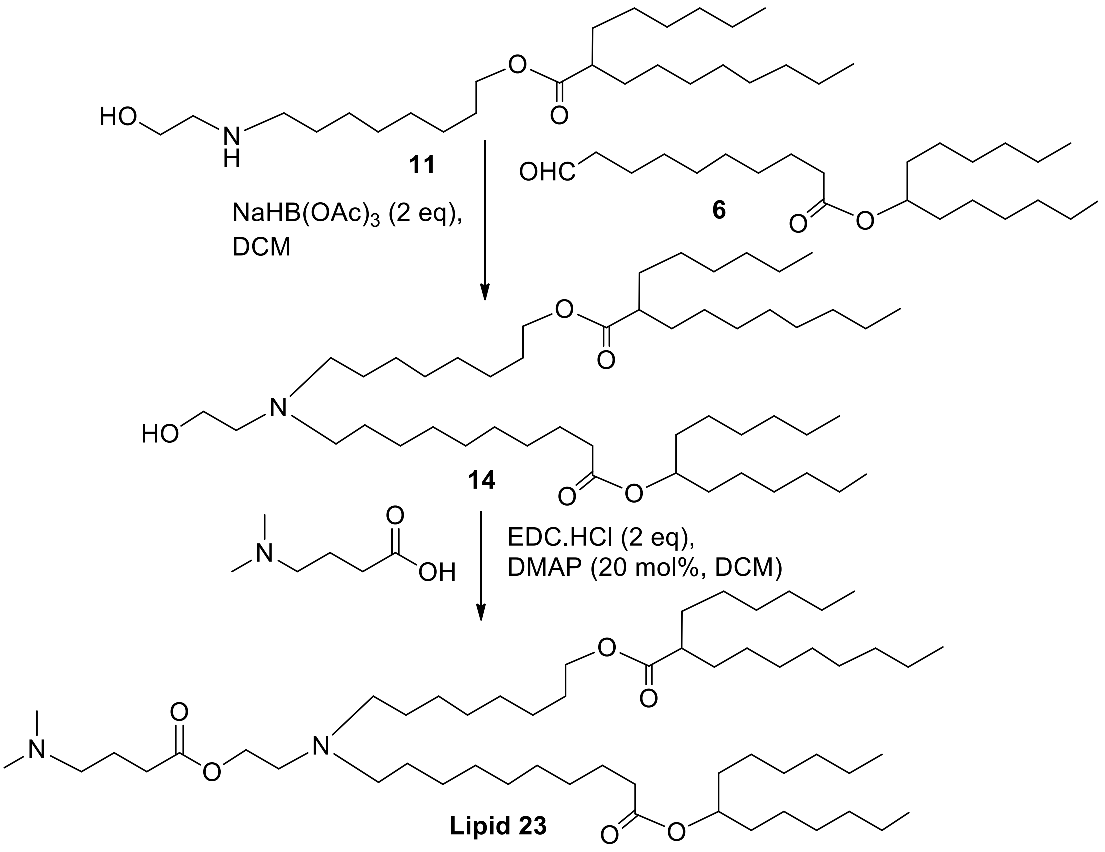

8-((2-hydroxyethyl)(10-oxo-10-(tridecan-7-yloxy)decyl)amino)octyl 2-hexyldecanoate

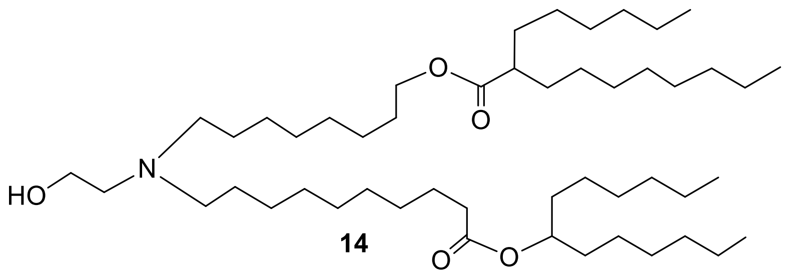

To the ethanolamine **11** (285 mg, 0.67 mmol, 1 equiv.), a solution of tridecan-7-yl 10-oxodecanoate **6** (294 mg, 0.80 mmol, 1.2 equiv.) in dry CH_2Cl_2 (20 mL) was added in under argon atmosphere and stirred for 2 hr at room temperature. Later sodium triacetoxyborohydride (281 mg, 1.33 mmol, 2.0 equiv.) was added and stirred overnight at the same temperature. Then the reaction was quenched with sat.$NaHCO_3$ solution followed by extract with CH_2Cl_2 (3 times). The organic layer was washed with brine solution and dried over anhydrous Na_2SO_4. The solvent was evaporated on a rotary evaporator, and the residue was purified by column chromatography using 0-5% Isopropanol in $CHCl_3$ to obtain the desired ethanolamine **14** (440 mg, 85%) as colorless liquid.

^{1}H NMR (400 MHz, $CDCl_3$): δ 4.86 (1 H, quint, J = 6.4 Hz), 4.06 (2 H, t, J = 6.8 Hz), 3.71-3.53 (2 H, br), 2.80-2.64 (2 H, br), 2.64-2.45 (4 H, br), 2.36-2.23 (1 H, m), 2.27 (2 H, t, J = 7.6 Hz), 1.83-1.54 (12 H, m), 1.54-1.38 (10 H, m), 1.38-1.03 (48 H, m), 0.87 (12 H, t, J = 7.2 Hz).

ESI-MS: *m/z* 781.1 $[M+1]^+$

8-((2-((4-(dimethylamino)butanoyl)oxy)ethyl)(10-oxo-10-(tridecan-7-yloxy)decyl)amino)octyl 2-hexyldecanoate

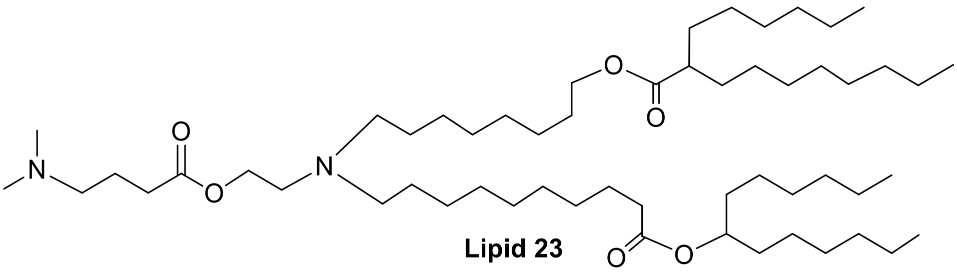

Lipid 23

The above ethanolamine **14** (440 mg, 0.56 mmol, 1 equiv.), *N, N*-dimethyl aminobutyric acid hydrochloride (188 mg, 1.12 mmol, 2 equiv.), EDC.HCl (214 mg, 1.12 mmol, 2 equiv.) and DMAP (14 mg, 0.11 mmol, 20 mol%) were dissolved in dry CH_2Cl_2 (20 mL) under an argon atmosphere and stirred overnight at room temperature. After that, the reaction was quenched with sat. $NaHCO_3$ followed by extract with CH_2Cl_2 (3 times). Then the organic portion was washed with brine solution and dried over anhydrous Na_2SO_4. The solvent was evaporated, and the residue was purified by column chromatography using 0-10% IPA in $CHCl_3$ to obtain **Lipid 23** (413 mg, 82%) as a colorless liquid.

^{1}H NMR (400 MHz, $CDCl_3$): δ 4.86 (1 H, quint, J = 6.4 Hz), 4.11 (2 H, t, J = 6.4 Hz), 4.06 (2 H, t, J = 6.8 Hz), 2.67 (2 H, t, J = 6.4 Hz), 2.43 (4 H, t, J = 7.6 Hz), 2.37-2.24 (3 H, m), 2.34 (2 H, t, J = 7.6 Hz), 2.27 (2 H, t, J = 7.6 Hz), 2.23 (6 H, s), 1.79 (2 H, quint, J = 7.6 Hz), 1.66-1.54 (6 H, m), 1.54-1.46 (4 H, m), 1.46-1.36 (6 H, m), 1.36-1.13 (54 H, m), 0.87 (12 H, t, J = 7.2 Hz).

ESI-MS: *m/z* 893.91 $[M+1]^+$; 915.9 $[M+Na]^+$

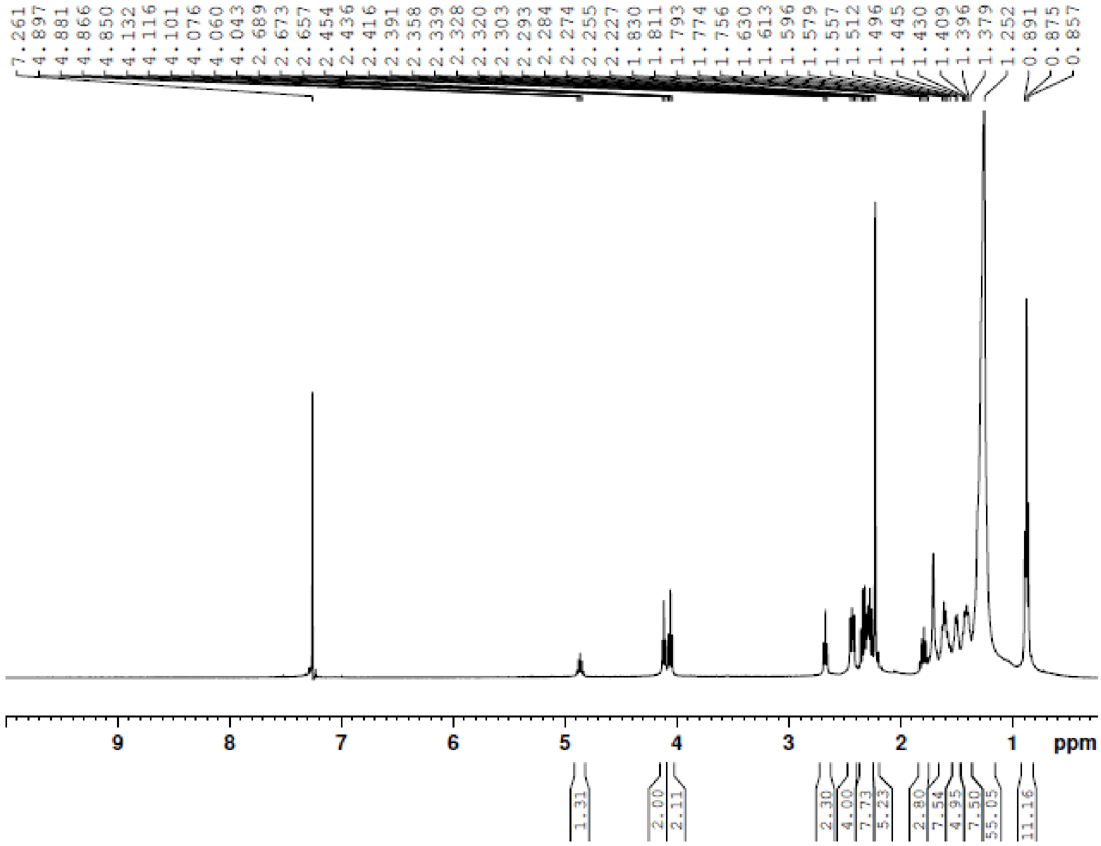
7.261
4.897
4.881
4.866
4.850
4.132
4.116
4.101
4.076
4.060
4.043
2.689
2.673
2.657
2.454
2.436
2.416
2.391
2.358
2.339
2.328
2.320
2.303
2.293
2.284
2.274
2.255
2.227
1.830
1.811
1.793
1.774
1.756
1.630
1.613
1.596
1.579
1.557
1.512
1.496
1.445
1.430
1.409
1.396
1.379
1.252
0.891
0.875
0.857
9
8
7
6
5
4
3
2
1
ppm
1.31
2.00
2.11
2.30
4.00
7.73
5.23
2.80
7.54
4.95
7.50
55.05
11.16

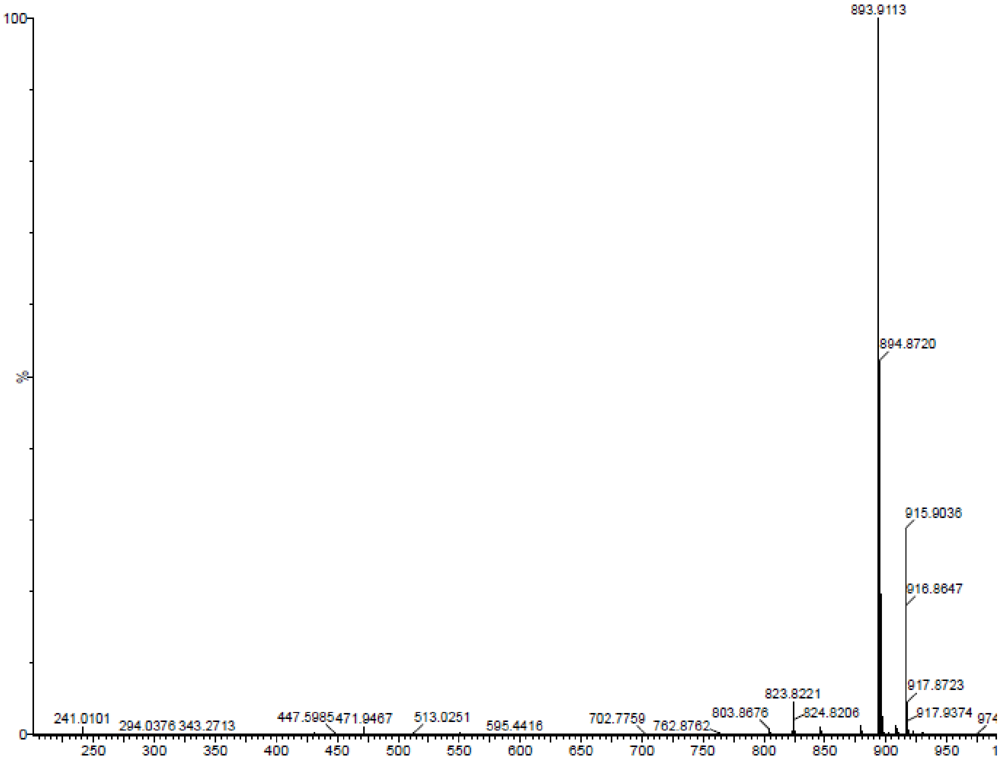
100
%
0
893.9113
894.8720
915.9036
916.8647
917.8723
917.9374
823.8221
824.8206
803.8676
762.8762
702.7759
595.4416
513.0251
447.5985
471.9467
343.2713
294.0376
241.0101
250
300
350
400
450
500
550
600
650
700
750
800
850
900
950

9. Synthesis of SM-102

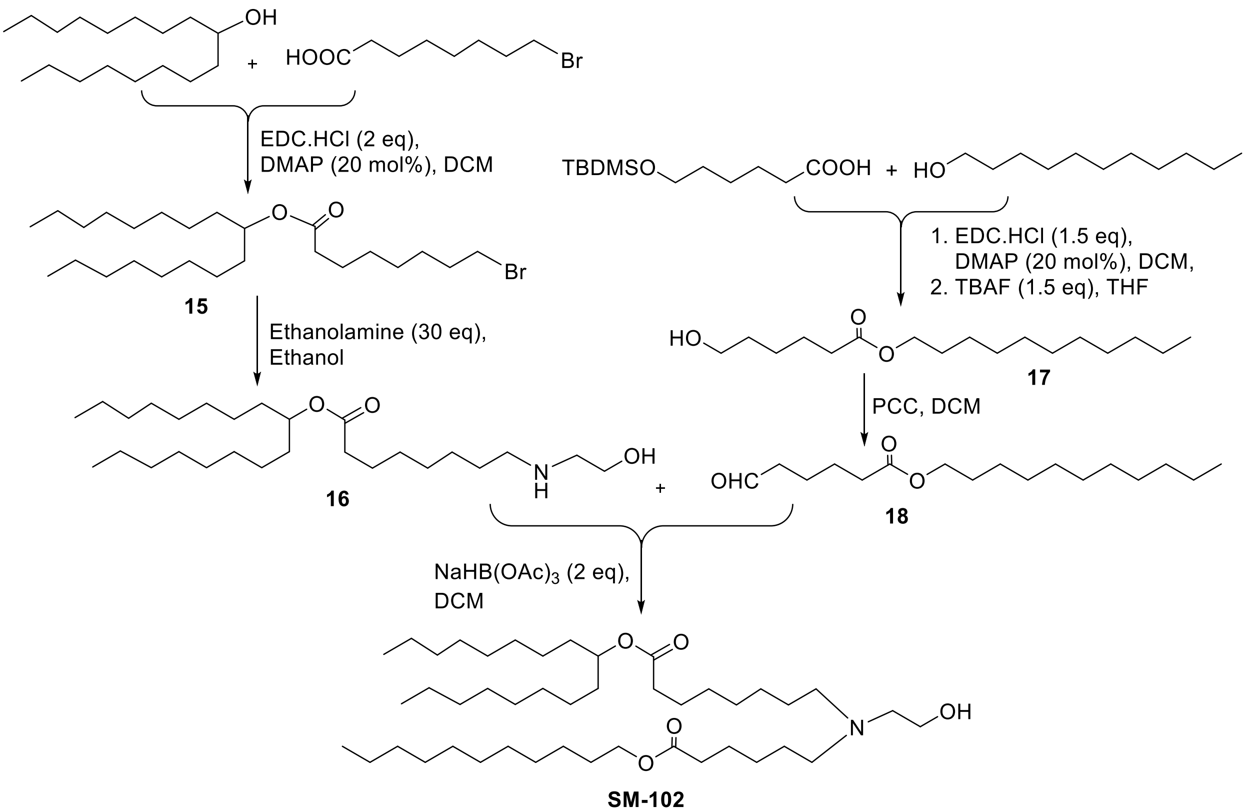

Heptadecan-9-yl 8-bromooctanoate

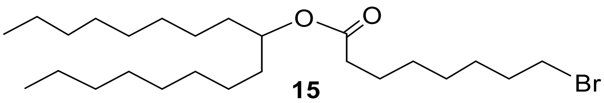

15

The heptadecan-9-ol (1.0 g, 3.90 mmol, 1 equiv.), 8-bromooctanoic acid (1.13 g, 5.08 mmol, 1.3 equiv.), EDC.HCl (1.50 g, 7.80 mmol, 2 equiv.) and DMAP (95 mg, 0.78 mmol, 20 mol%) were dissolved in dry CH_2Cl_2 (30 mL) under an argon atmosphere and stirred overnight at room temperature. After that, the reaction was quenched with sat. $NaHCO_3$ followed by extract with CH_2Cl_2 (3 times). Then the organic portion was washed with brine solution and dried over anhydrous Na_2SO_4. The solvent was evaporated, and the residue was purified by column chromatography using 0-5% EtOAc in hexane to get the desired bromide **15** (1.3 g, 72%) as a colorless liquid.

^{1}H NMR (400 MHz, $CDCl_3$): δ 4.86 (1 H, quint, J = 4.8 Hz), 3.52 (1 H, t, J = 6.8 Hz), 3.40 (1 H, t, J = 6.8 Hz), 2.28 (2 H, t, J = 7.6 Hz), 1.85 (1 H, quint, J = 7.6 Hz), 1.77 (1 H, quint, J = 7.6 Hz), 1.71-1.38 (10 H, m), 1.38-1.14 (26 H, m), 0.88 (6 H, t, J = 7.2 Hz).

Heptadecan-9-yl 8-((2-hydroxyethyl)amino)octanoate

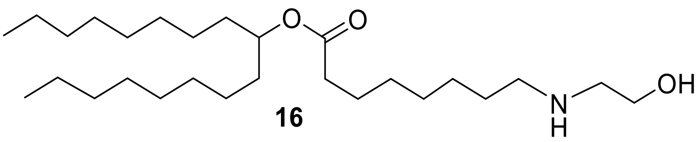

16

The above bromide **15** (1.26 g, 2.73 mmol, 1 equiv.), and ethanolamine (4.95 mL, 82 mmol, 30 equiv.) were dissolved in ethanol (5 mL) and stirred overnight at 65 ºC. After that, the solvent was evaporated under reduced pressure, then the reaction mixture was poured into the water and extracted with ethyl acetate (3 times). The organic portion was washed with brine solution and dried over with anhydrous Na_2SO_4. The solvent was evaporated, and the residue was purified by column chromatography using 0-15% Methanol (1% triethylamine) in Chloroform to get the desired ethanolamine **16** (1.0 g, 83%) as a colorless liquid.

^{1}H NMR (400 MHz, $CDCl_3$): δ 4.86 (1 H, quint, J = 6.4 Hz), 3.64 (2 H, t, J = 5.2 Hz), 2.78 (2 H, t, J = 5.2 Hz), 2.62 (2 H, t, J = 7.2 Hz), 2.28 (2 H, t, J = 7.6 Hz), 1.69-1.56 (4 m, H), 1.55-1.42 (8 H, m), 1.40-1.16 (26 H, m), 0.88 (6 H, t, J = 7.2 Hz).

ESI-MS: *m/z* 442.7 $[M+1]^+$

Undecyl 6-hydroxyhexanoate

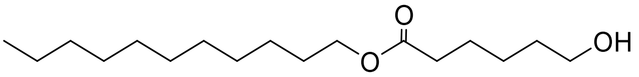

17

The 6-((tert-butyldimethylsilyl)oxy)hexanoic acid (4.0 g, 16.26 mmol, 1 equiv.), undecanol (4.04 mL, 19.51 mmol, 1.2 equiv.), EDC.HCl (4.66 g, 24.39 mmol, 1.5 equiv.) and DMAP (297 mg, 2.44 mmol, 15 mol%) were dissolved in dry CH_2Cl_2 (80 mL) under an argon atmosphere and stirred overnight at room temperature. After that, the reaction was quenched with sat. $NaHCO_3$ followed by extract with CH_2Cl_2 (3 times). Then the organic portion was washed with brine solution and dried over with anhydrous Na_2SO_4. The solvent was evaporated, and the residue was purified by column chromatography using 0-5% EtOAc in hexane to obtain the desired silyl product (6.1 g, 94%) as a colorless liquid.

To a solution of silyl product (6.1 g, 15.25 mmol, 1 equiv.) in THF (30 mL), TBAF (23 mL, 1.0 M in THF, 22.87 mmol, 1.5 equiv.) was added and stirred for 3 hr at room temperature. After that, the reaction was quenched with sat. NH_4Cl solution and extracted with 20% ethyl acetate in diethyl ether (3 times). The solvent was evaporated under reduced pressure, and the residue was purified by column chromatography using 0-20% EtOAc in hexane to obtain undecyl 6-hydroxyhexanoate **17** (4.0 g, 92%) as a colorless liquid.

^{1}H NMR (400 MHz, $CDCl_3$): δ 4.06 (2 H, t, J = 6.8 Hz), 3.65 (2 H, t, J = 5.6 Hz), 2.31 (2 H, t, J = 7.6 Hz), 1.74-1.51 (6 H, m), 1.47-1.17 (18 H, m), 0.88 (3 H, t, J = 6.8 Hz).

Undecyl 6-oxohexanoate

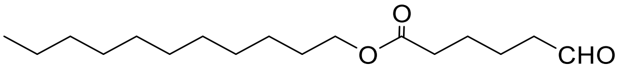

18

To a solution, undecyl 6-hydroxyhexanoate **17** (1.0 g, 3.5 mmol, 1 equiv.) in dry CH_2Cl_2 (30 mL), molecular sieves (4Å MS) were added under argon atmosphere, and then PCC (1.5 g, 7.0 mmol, 2 equiv.) was added portion wise and stirred for 2 hr. Afterward, the reaction mixture was filtered through a silica gel column to remove PCC, followed by a wash with 20% ethyl acetate in hexane (2 × 100 mL). The solvent was evaporated under reduced pressure to obtain the undecyl 6-oxohexanoate **18** (0.85 g, 85%) as a colorless liquid.

^{1}H NMR (400 MHz, $CDCl_3$): δ 9.77 (1 H, t, J = 1.6 Hz), 4.06 (2 H, t, J = 6.8 Hz), 2.50-2.41 (2 H, m), 2.40-2.26 (2 H, m), 1.79-1.52 (6 H, m), 1.40-1.15 (16 H, m), 0.88 (3 H, t, J = 7.2 Hz).

Heptadecan-9-yl 8-((2-hydroxyethyl)(6-oxo-6-(undecyloxy)hexyl)amino)octanoate

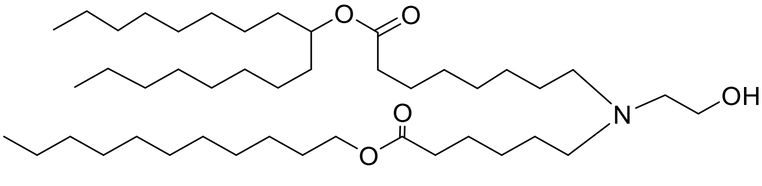

SM-102

To the ethanolamine **16** (320 mg, 0.72 mmol, 1 equiv.), a solution of undecyl 6-oxohexanoate **18** (247 mg, 0.87 mmol, 1.2 equiv.) in dry CH_2Cl_2 (20 mL) was added in under argon atmosphere and stirred for 2 hr at room temperature. Later sodium triacetoxyborohydride (305 mg, 1.45 mmol, 2.0 equiv.) was added and stirred overnight at the same temperature. Then the reaction was quenched with sat. $NaHCO_3$ solution followed by extract with CH_2Cl_2 (3 times). The organic layer was washed with brine solution and dried over anhydrous Na_2SO_4. The solvent was evaporated on a rotary evaporator, and the residue was purified by column chromatography using 0-5% Isopropanol in $CHCl_3$ to obtain the desired product **SM-102** (498 mg, 97%) as a colorless liquid.

^{1}H NMR (400 MHz, $CDCl_3$): δ 4.86 (1 H, quint, J = 6.0 Hz), 4.05 (2 H, t, J = 6.8 Hz), 3.53 (2 H, t, J = 7.6), 2.68-2.54 (2 H, br), 2.54-2.38 (4 H, br), 2.30 (2 H, t, J = 7.6 Hz), 2.27 (2 H, t, J = 7.6 Hz), 1.69-1.55 (6 H, m), 1.55-1.38 (10 H, m), 1.38-1.08 (46 H, m), 0.88 (3 H, t, J = 5.6

Hz), 0.87 (6 H, t, $J = 7.2$ Hz).

ESI-MS: m/z 710.9 $[M+1]^+$

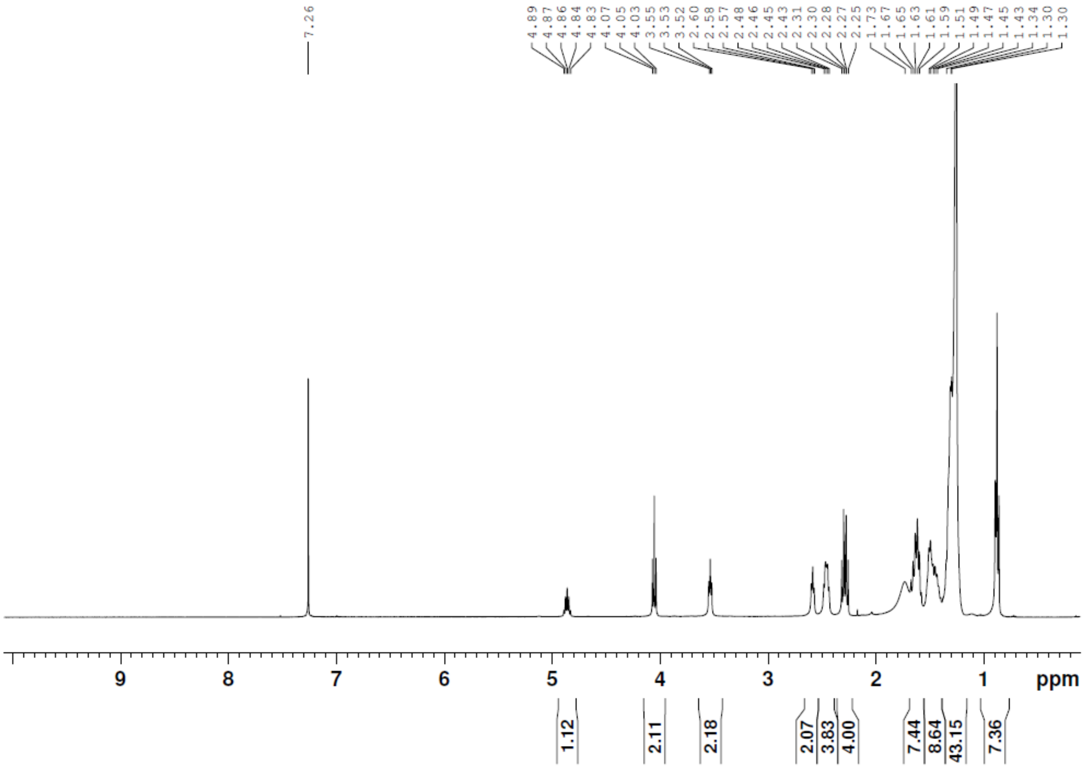

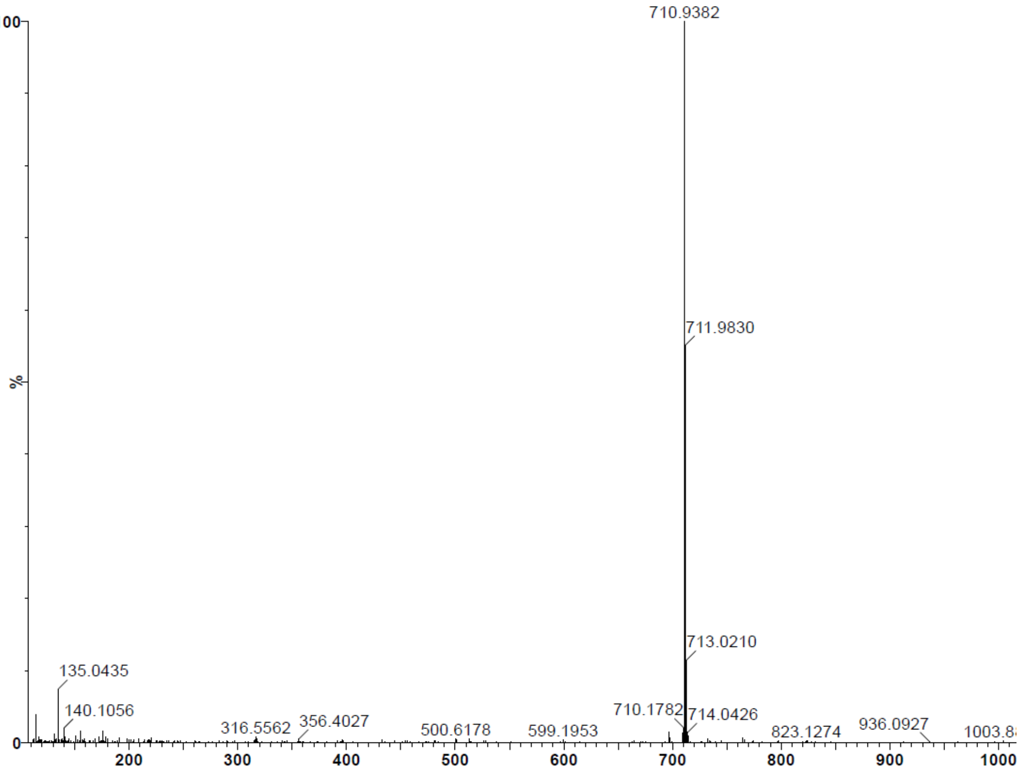

Cell lines.

Murine melanoma, B16F10 (ATCC®, CRL-6475™), colorectal carcinoma cancer cell, CT26 (ATCC®, CRL-2638™) and murine macrophage cell, Raw 264.7 (ATCC®, TIB-71™) were maintained in DMEM, and RPMI1640 (Gibco, Thermo-Fisher Scientific, Inc) supplemented with FBS (10%, Biological Industries, Israel), L-Glutamine (1%, Gibco, Thermo-Fisher Scientific, Inc), Penicillin-Streptomycin-Nystatin (1%, Biological Industries, Israel). Cells were detected for Mycoplasma contamination using EZ-PCR Mycoplasma Test Kit (Biological Industries, Israel) and were passaged every 2 to 3 days.

mRNAs.

Chemically modified mRNAs encoding firefly luciferase (62kD), mCherry (28kD), and Cre recombinase (39kD) were provided by BioNTech or purchased from TriLink®.BioTechnologies.

Lipid nanoparticle preparation.

All ionizable lipid was synthesized as described in the lipid synthesis section. PEG-DMG, DSPC, and Cholesterol were purchased from Avanti Polar Lipids Inc. 1 volume of lipid (Ionizable lipid, DSPC, DMG-PEG, Cholesterol at 40:10:1.5:48.5 mol ratio in EtOH). Three volumes of mRNA (lipid to mRNA, weight ratio 27:1) in a sodium acetate buffer (pH4.5) were mixed through a microfluidic mixing device Nanoassemblr (Precision Nanosystems Inc) at a flow rate of 12 ml min^{-1}. The prepared mRNA-LNPs were dialyzed against phosphate-buffered saline (PBS, pH 7.4) for 20-24hr.

RNA encapsulation and quantification.

The Quant-iT RiboGreen RNA assay kit (Life Technologies) was used to measure the mRNA encapsulation in LNPs. In brief, 0.5 μL of LNP was diluted in a final volume of 100 μL of TE buffer (20 mᴍ EDTA, 10 mᴍ Tris-HCL) with or without Triton X-100 (0.5%, Sigma-Aldrich).

Samples were loaded in a 96-well black plate (Costar, Corning). The plate was incubated for 15 min at 37 °C before adding 100 μL of TE buffer (0.5% v/v, RiboGreen reagent) to each well. The fluorescence was detected using a microplate reader (Biotek Industries) according to the manufacturer's protocol.

Size and ζ-potential analysis of LNPs.

Nano size and ζ-potential of prepared mRNA-LNPs were analyzed by dynamic light scattering (DLS) using a Malvern nano ZS ζ-sizer (Malvern Instruments). Briefly, mRNA-LNPs were diluted in double-distilled water (1:50, volume ratio) and PBS (1:50, volume ratio) for ζ-potential and size measurements, respectively.

In vitro luciferase assay.

B16F10, CT26, and Raw 264.7 cells were seeded ($3x10^4$ cells mL^{-1}) in 96 well tissue culture plates (Greiner bio-one, Germany). At 24hr post-seeding, cells were treated with mRNA-LNPs (firefly luciferase mRNA, 0.2 or 0.4 μg mL^{-1}). At 24hr post-LNP treatment, cells were lysed, and luciferase activity was measured using the Promega® Luciferase assay kit according to the manufacturer's protocol.

TNS assay.

As previously described[2], the pKa values of LNPs were measured using the 2-(p-toluidino)-6-naphthalenesulfonic acid (TNS) assay. In brief, the master buffer was prepared using 10 mM 4-(2-hydroxyethyl)-1-piperazineethanesulfonic acid (HEPES), 10 mM 4-morpholineethanesulfonic acid (MES), 10 mM ammonium acetate, and 130 mM sodium chloride (NaCl). Sixteen buffers with a pH ranging from 2.5 to 10 were prepared using 1.0 M sodium hydroxide and 1.0 M hydrochloric acid based on the master buffer. 6-(p-toluidino)-2-naphthalenesulfonic acid sodium salt (TNS reagent) was prepared as a 0.1 mM stock solution in Milli-Q water. 90 μL of each buffer was added in triplicate to a black 96-well plate, and then

6 µL of 0.1 mM total lipid LNPs were added to each well. After that, 5 µL of TNS stock solution was added to each well and kept on the shaker to mix properly for 10 min by covering the plate with aluminum foil. Fluorescence intensity was measured using excitation and emission wavelengths 322 nm and 431 nm, respectively. A pKa curve was plotted by taking the pH values on the x-axis and the normalized fluorescence values on the y-axis.

Animal experiments.

All animal protocols were approved by the Tel Aviv University, Institutional Animal Care and Usage Committee (IACUC) in accordance with current regulations and standards of the Israel Ministry of Health. All animal experiments were double-blinded; the researchers were blinded to group allocation and administered treatments. Mice were randomly divided in a blinded fashion at the beginning of each experiment. Pre-established criteria for removing animals from the experiments were based on animal health, behavior, and well-being as required by ethical guidelines.

In vivo luciferase assay & organ distribution study.

Eight to ten weeks-old female C57BL/6 mice (Envigo, Rehovot, Israel) were intravenously injected with mLuc-LNPs (mRNA dose: 0.5 mg kg^{-1}). At 6 and 24hr post-injection, mice were intraperitoneally injected with D-Luciferin (150 mg kg^{-1}), and major organs were harvested for imaging using IVIS-spectrum-CT (Perkin Elmer Inc).

In vivo mCherry mRNA delivery & cellular expression study.

Eight to ten weeks-old female C57BL/6 mice were intravenously injected with mCherry mRNA-LNPs (mRNA dose: 0.5 mg kg^{-1}). At 6hr post-injection, mice were sacrificed to harvest organs and single-cell digestion for flow cytometry analysis.

In vivo Cre recombinase mRNA delivery & cellular tdTomato-expression study.

Twelve to sixteen weeks-old female Cre mice (Ai9(RCL-tdT), The Jackson Laboratory) were

intravenously injected with Cre mRNA-LNPs (mRNA dose: 0.6 mg kg^{-1}). At 72hr post-injection, mice were sacrificed to harvest organs for flow cytometric analysis.

Flow cytometry analysis of organ cells.

Single cells were prepared via organ digestion (Collagenase type IV, 0.5 mg mL^{-1} in PBS, 5 ml) and filtered through a cell strainer (100 µm pore size). After red blood cell lysis, cells were washed three times with cold PBS and stained with antibodies (CD45, CD31, CD11b, CD11c, F4/80, Gr1, CD3, CD19) for flow cytometry analysis (Cytoflex™ and the Cytexpert™ software, Beckman-Coulter, USA.).

In vivo toxicity study.

Eight to ten weeks-old female C57BL/6 mice were intravenously injected with mLuc-LNPs (mRNA dose: 0.5 mg kg^{-1}) for toxicity analysis. At 24hr post-injection, mice were sacrificed to harvest whole blood from the heart. Blood was stored at RT for 30 min and centrifuged to separate serum. Samples were sent to American Medical Laboratories (AML) for blood chemistry analysis. The spleen and liver were fixed with 4% formaldehyde and sent to Histospeck for H&E staining and analysis.

Immunogenicity study.

Eight to ten weeks-old female C57BL/6 mice were intravenously injected with mLuc-LNPs (mRNA dose: 0.5 and 1.0 mg kg^{-1}). At 3 and 24hr post-injection, mice were sacrificed to collect whole blood from the heart. Blood was stored at RT for 30 min and centrifuged to separate serum. Samples were diluted, and measured cytokine levels using ELISA kits (ab100721, ab157711).

Statistical Analysis.

All statistical analysis were performed using one-way ANOVA with Tukey's post-hoc test in GraphPad Prism 7 Project software. All data are presented as mean ± standard deviation (S.D.)

and standard error of the mean (S.E.M.). *P<0.05; **P<0.01, ***P<0.001.

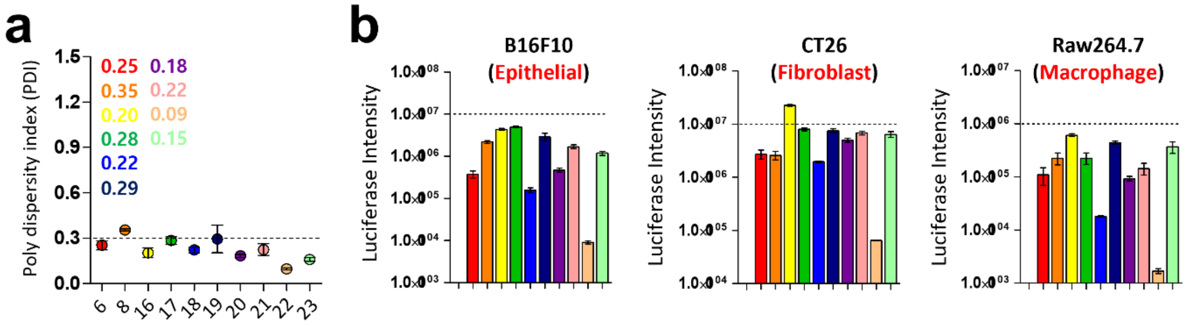

Supplementary Figure 1. PDI value for LNPs & *in vitro* luciferase assay in non-hepatic cell lines. (a) The polydispersity index (PDI) of mLuc-LNPs at 1-day post-preparation was shown. Numbers indicate the average PDI value for each LNP. Data are shown as mean ± S.D., n=3-6/group. **(b)** Bar graph for *in vitro* luciferase assay. Cells were treated with firefly luciferase mRNA-LNPs (mRNA conc: 0.2 μg mL^{-1} and luciferase activity was measured at 24hr post-treatment. This result was converted to the Heat map image (Origin 2022b) in Figure 2g. Data are shown as mean ± S.E.M., n=3-6/group.

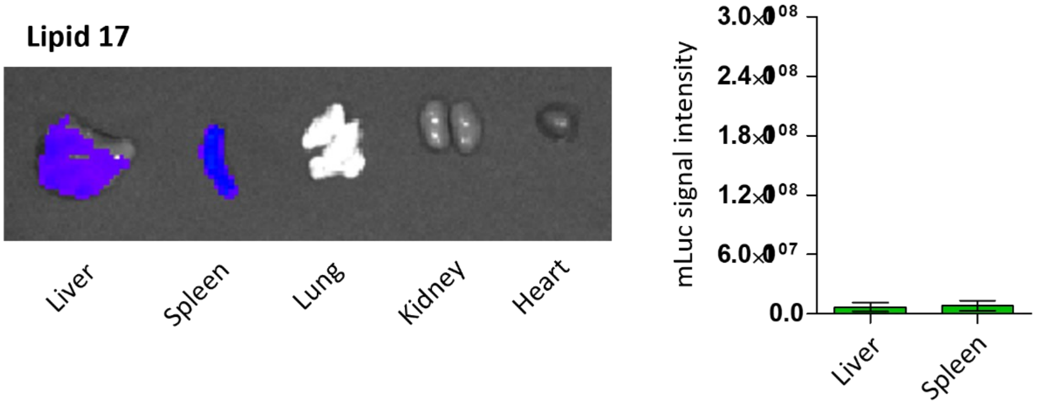

Supplementary Figure 2. In vivo luciferase assay with the lipid 17 LNP.

Mice were intravenously injected with mLuc-LNP 17 (mRNA dose: 0.5 mg kg^{-1}), and luciferase activity was measured in organs at 6hr post-injection. Data are shown as mean ± S.D., mice n=3.

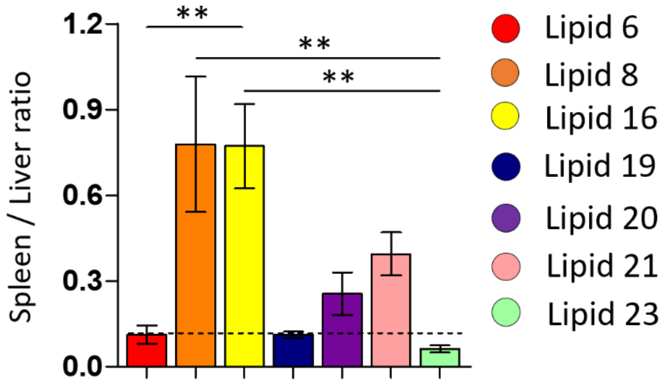

Supplementary Figure 3. Organ luciferase activity ratio between spleen and liver.

In vivo, the luciferase activity ratio of mRNA-LNPs was shown (At 6hr post-injection). Data are shown as mean ± S.E.M., ***P*<0.01 by one-way ANOVA with Tukey's post hoc test, mice n=3-5/group.

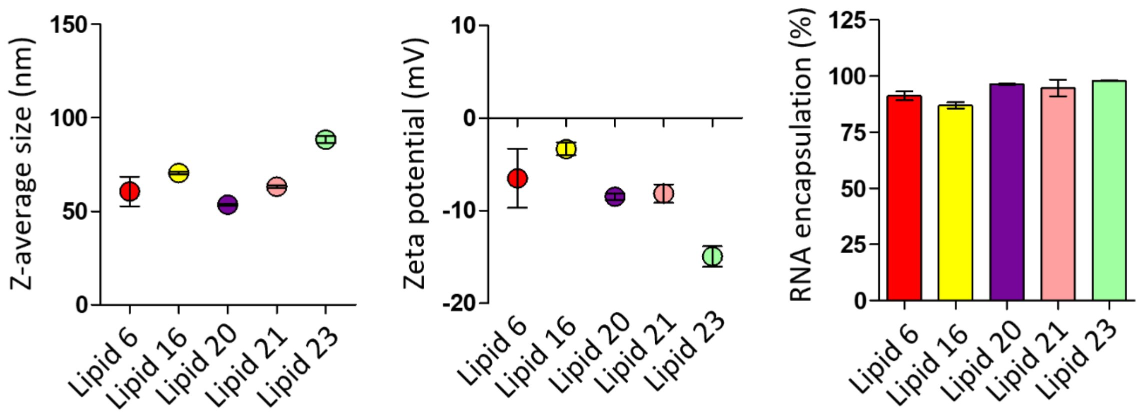

Supplementary Figure 4. Physicochemical characterization of mCherry mRNA-LNPs.

Nano-size and zeta-potential of mCherry mRNA-LNPs were analyzed by dynamic light scattering (DLS). RNA encapsulation was analyzed by calculating the percentile of LNP-encapsulated RNA in preparations. Data are shown as mean ± S.D., n=3/group.

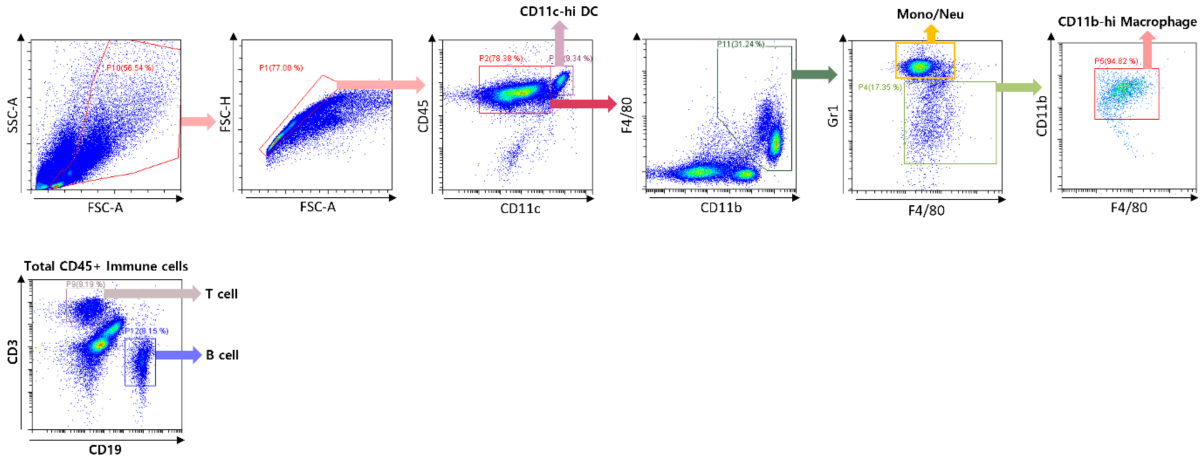

Supplementary Figure 5. Gating strategy for flow cytometric analysis of organ macrophages and immune cells.

Total single cells were stained with antibodies (CD45, CD11b, CD11c, F4/80, Gr1, CD3, CD19 and CD31) and gated for flowcytometric analysis. Representative plots from the cells of lung tissue were shown.

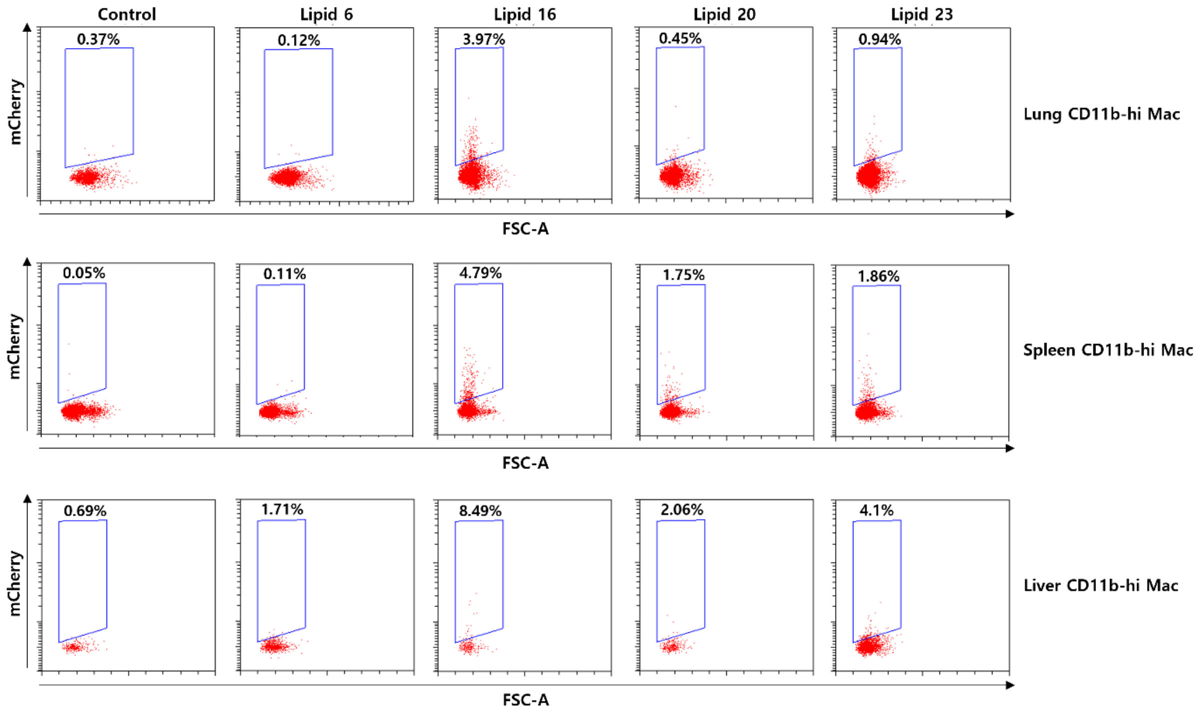

Supplementary Figure 6. mCherry-expression for CD11b^{hi} organ macrophages.

Mice were injected with mCherry mRNA-LNPs (mRNA dose: 0.5 mg kg^{-1}), and mCherry-expression in organ macrophages was measured at 6hr post-injection. Representative plots were shown.

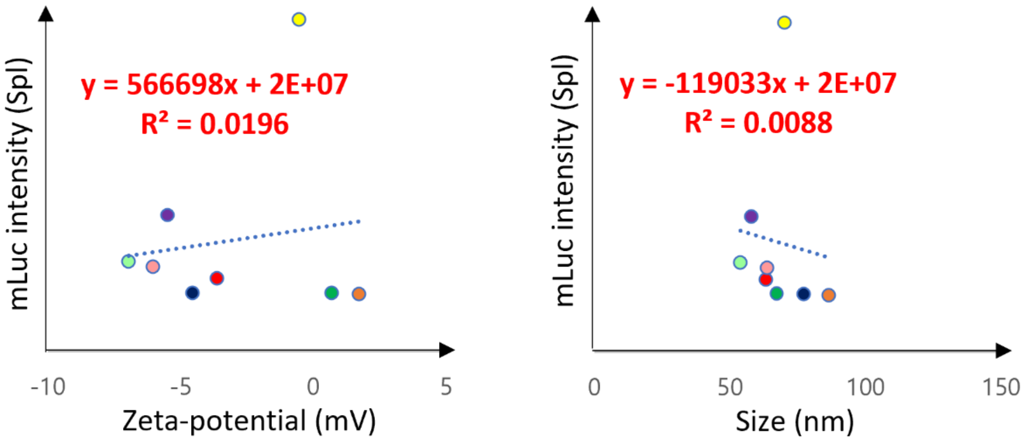

Supplementary Figure 7. Correlation study for splenic mRNA delivery and physicochemical characteristic of LNPs.

Correlation between nano-size, ζ-potential of LNPs, and spleen mLuc expression was shown. Each dot indicates mean values of spleen luciferase intensity, Zeta-potential, and Size of LNPs

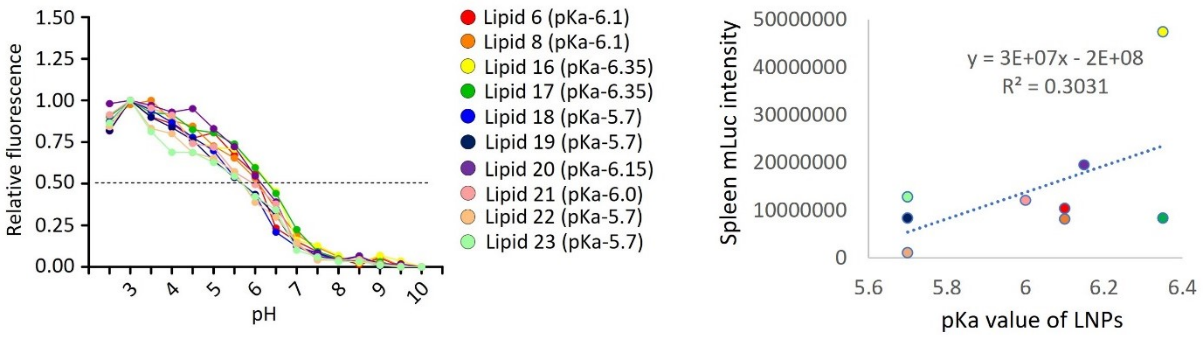

Supplementary Figure 8. pKa value of the LNPs & correlation with the splenic mLuc-expression.

pKa values of the mRNA-encapsulated LNPs were evaluated by TNS assay. pKa values were calculated based on the 3-independent experiments.

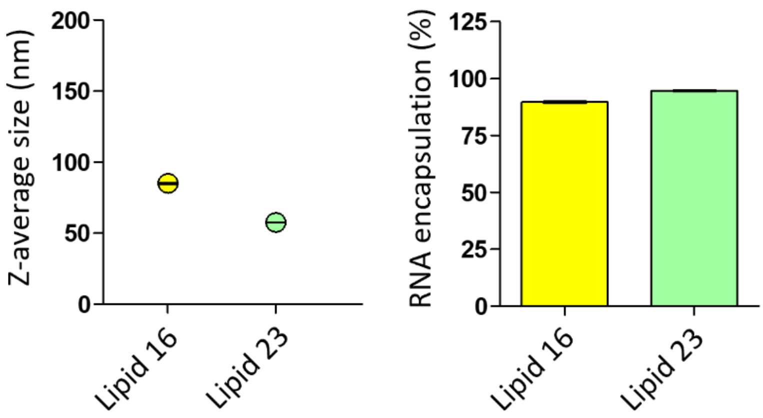

Supplementary Figure 9. Physicochemical characterization of Cre recombinase mRNA-LNPs.

The Cre mRNA-LNPs were prepared with Lipids 16 and 23 and analyzed by DLS. RNA encapsulation was analyzed by calculating the percentile of LNP-encapsulated RNA in preparations. Data are shown as mean ± S.D., n=3/group.

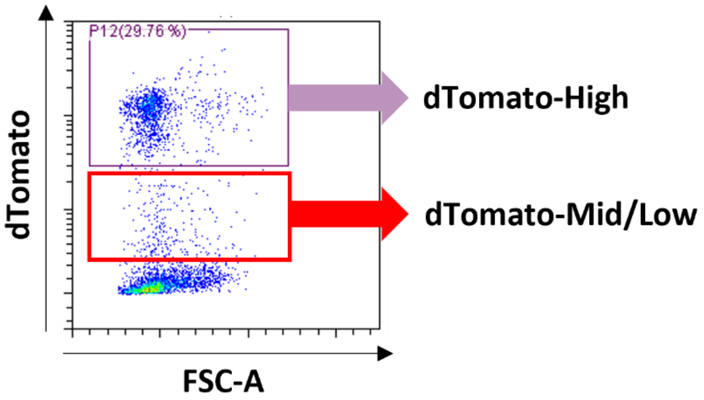

Supplementary Figure 10. Gating strategy for tdTomato-expression in Cre mRNA-LNP study.

The tdTomato^{-High} cells were gated as Cre mRNA-delivered cells.

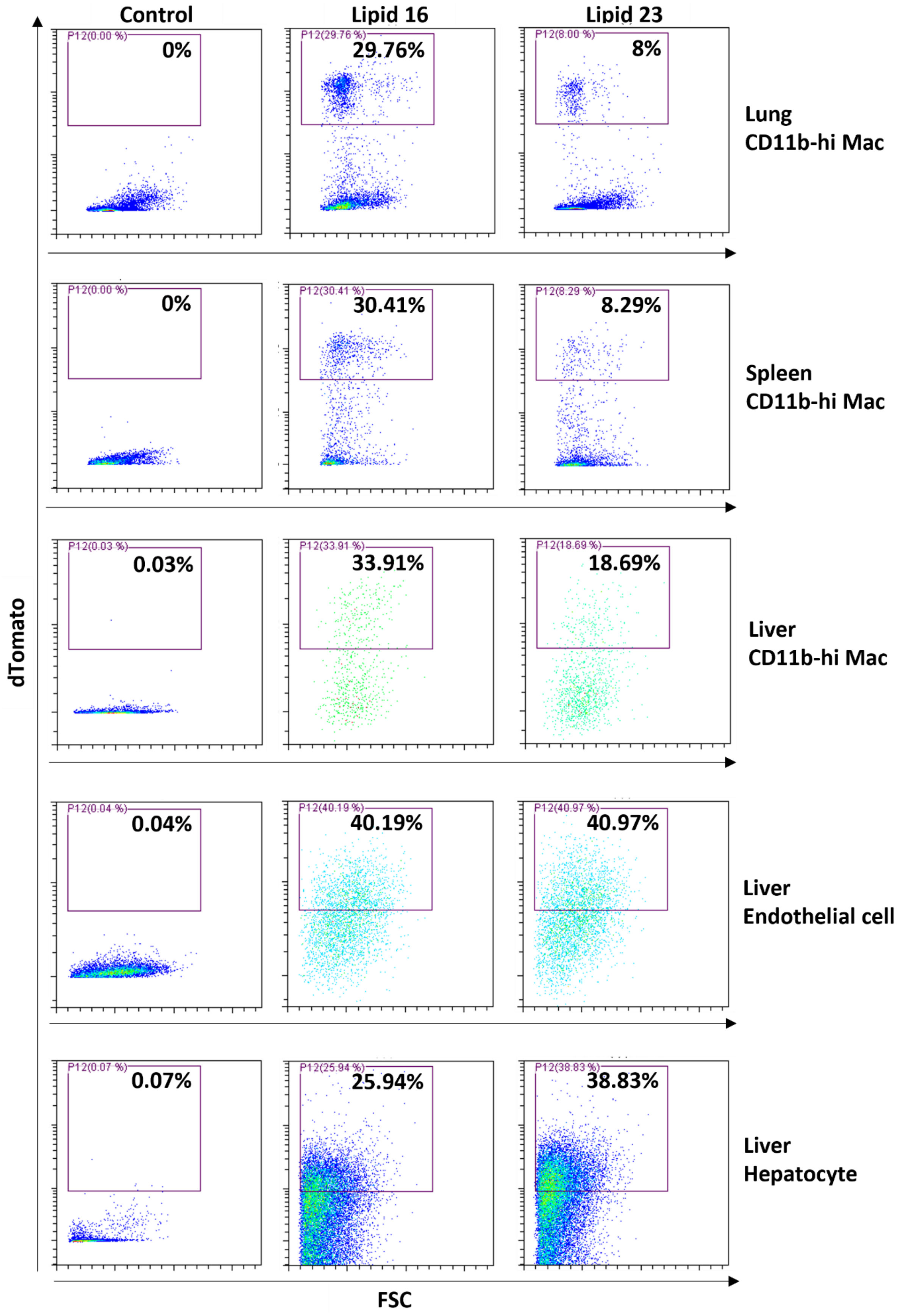

Supplementary Figure 11. The Cre mRNA-mediated tdTomato-expression in organ

macrophages and hepatocytes.

Representative plots for tdTomato-expression in organ macrophages, liver endothelial cells, and hepatocytes were shown. The mice were injected with Cre mRNA-LNPs, and organs were harvested at 72hr post-injection to measure tdTomato-expression.

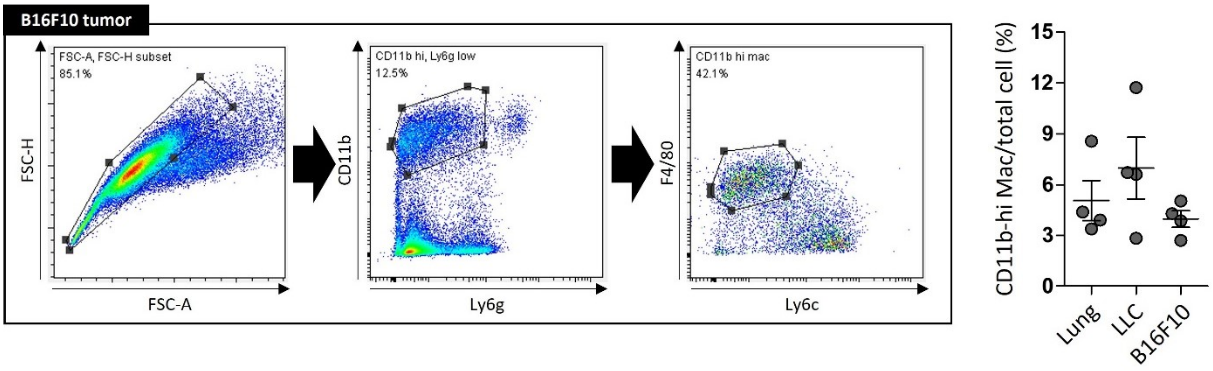

Supplementary Figure 12. Percentile of the CD11b^{hi} macrophages in B16F10 & LLC tumors.

C57BL/6 mice were injected with B16F10 (melanoma) or LLC (lung cancer) cells on the hind leg, and tumors were harvested for flow cytometric analysis when the tumor size reached 200-400 mm^3. CD11b^{hi} Ly6g$^{mid/low}$ Ly6c$^{mid/low}$ F4/80$^+$ cells were gated as CD11b^{hi} macrophages in the tumor. Data are presented as mean ± S.E.M., mice n=4/group.

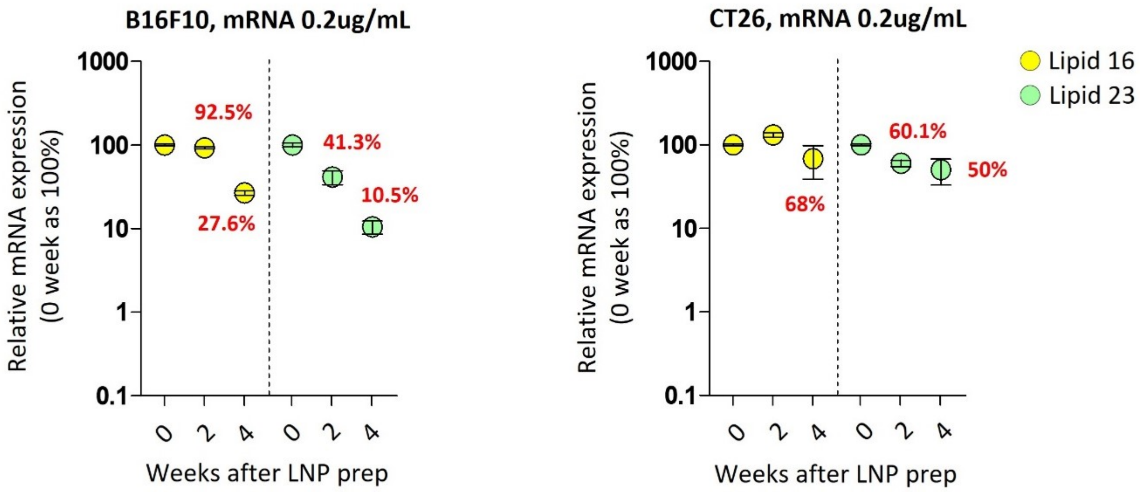

Supplementary Figure 13. mRNA delivery potency of the LNPs *in vitro*.

Lipid 16- and 23-LNPs were treated on the B16F10 and CT26 cells every two weeks to evaluate mRNA delivery potency (mLuc concentration: 0.2ug mL^{-1}). Data are presented as mean ± S.E.M., n=6-7/group from 2-independent experimental sets. Luciferase intensity was normalized to that of 0-week results.

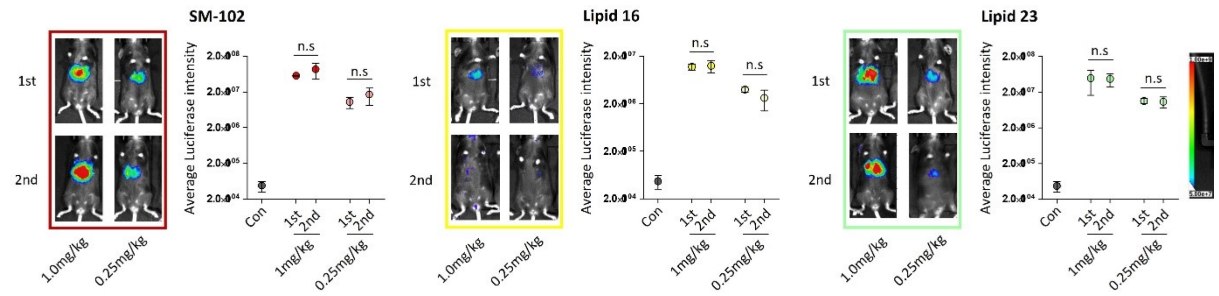

Supplementary Figure 14. Tolerability of the mRNA-LNPs for repeated dosing.

C57BL/6 mice were injected with mLuc-LNPs two times (at 4-day intervals), and luciferase intensity was measured at 6hr-post injection (dose: 0.25 and 1 mg kg^{-1}). Data are presented as mean ± S.E.M., 'n.s' indicates 'not significant' by one-way ANOVA with Tukey's post hoc test, mice n=2-3/group.

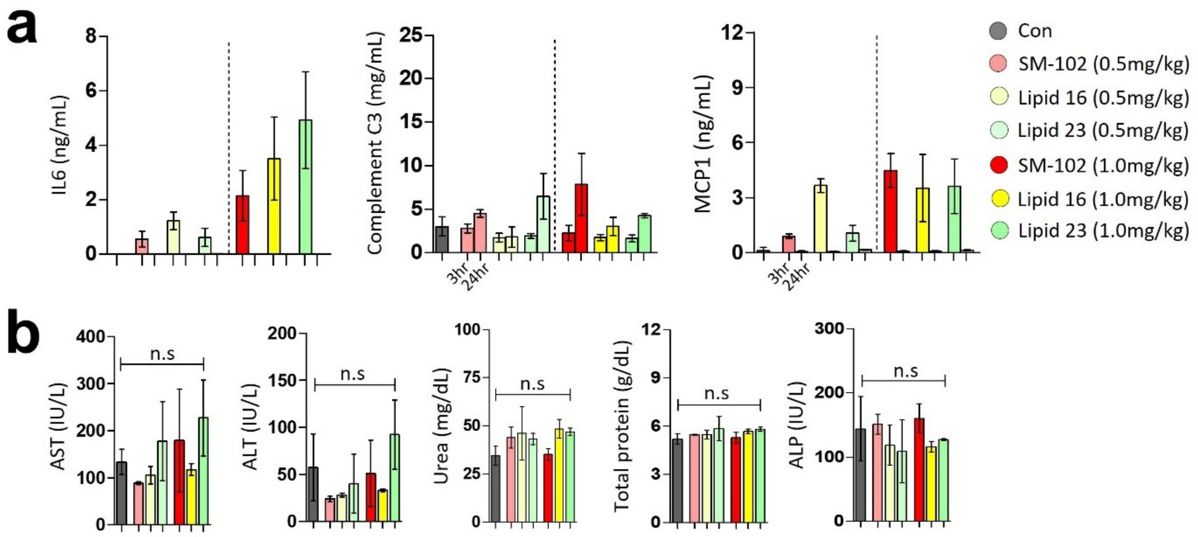

Supplementary Figure 15. Immunogenicity & toxicity of the mRNA-LNPs in comparison with SM-102 LNP.

C57BL/6 mice were injected with the Lipid 16-, 23-, and SM-102 LNPs, and blood was collected at 3 and 24hr-post injection (dose: 0.5, 1.0 mg kg^{-1}). **a)** ELISA results of the blood cytokine level for immunogenicity. **b)** Liver enzymes & blood chemistry results. Data are presented as mean ± S.D., n.s= not significant, mice n=2-3/group.

[1] S. Ramishetti, I. Hazan-Halevy, R. Palakuri, S. Chatterjee, S. Naidu Gonna, N. Dammes, I. Freilich, L. Kolik Shmuel, D. Danino, D. Peer, *Advanced Materials* **2020**, 1906128.

[2] S. Liu, Q. Cheng, T. Wei, X. Yu, L. T. Johnson, L. Farbiak, D. J. Siegwart, *Nature materials* **2021**, *20*, 701-710.
